# A Dimeric Thiourea CSA for the Enantiodiscrimination of Amino Acid Derivatives by NMR Spectroscopy

**DOI:** 10.1021/acs.joc.1c00340

**Published:** 2021-05-21

**Authors:** Alessandra Recchimurzo, Cosimo Micheletti, Gloria Uccello-Barretta, Federica Balzano

**Affiliations:** Department of Chemistry and Industrial Chemistry, University of Pisa, via Moruzzi 13, 56124 Pisa, Italy

## Abstract

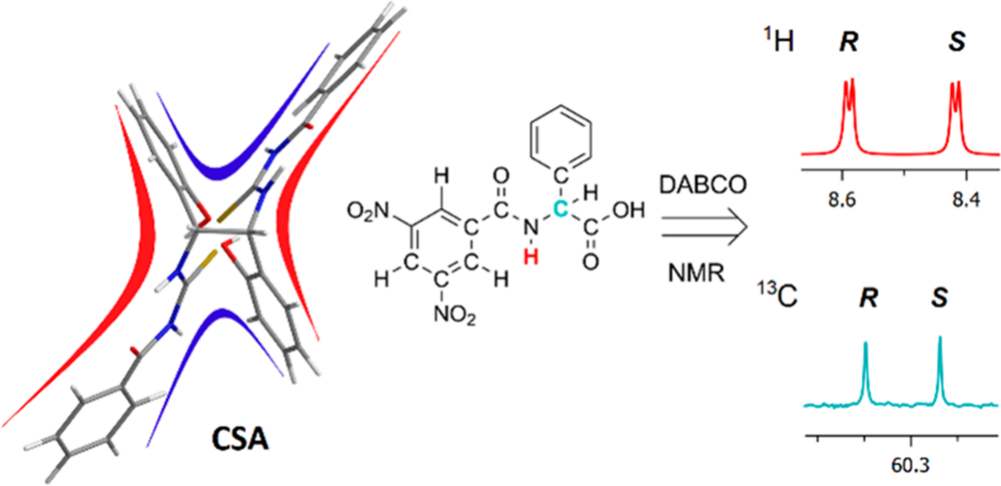

The reaction of benzoyl isothiocyanate with (1*R*,2*R*)-1,2-bis(2-hydroxyphenyl)ethylenediamine afforded a new thiourea chiral solvating agent (CSA) with a very high ability to differentiate ^1^H and ^13^C NMR signals of simple amino acid derivatives, even at low concentrations. The enantiodiscrimination efficiency was higher with respect to that of the parent monomer, a thiourea derivative of 2-((1*R*)-1-aminoethyl)phenol, thus putting into light the relevance of the cooperativity between the two molecular portions of the dimer in a cleft conformation stabilized by interchain hydrogen bond interactions. An achiral base additive (DABCO or DMAP) played an active role in the chiral discrimination processes, mediating the interaction between the CSA and the enantiomeric mixtures. The chiral discrimination mechanism was investigated by NMR spectroscopy through the determination of complexation stoichiometries, association constants, and the stereochemistry of the diastereomeric solvates.

## Introduction

Oftentimes, dramatic differences in the pharmacodynamics and pharmacokinetics of pure enantiomeric forms of therapeutic agents have increasingly brought to light the outstanding role of chirality and a generated great awareness of the need for rigorous and reproducible methods to properly identify and quantify stereoisomeric forms of chiral substrates, with a strong preference toward noninvasive methods involving minimum manipulative procedures, such as spectroscopic methods. Among them, nuclear magnetic resonance (NMR) spectroscopy is notable because it provides several measurable parameters for each of the observable NMR-active nuclei of the enantiomeric products as long as they are made intrinsically anisochronous and hence differentiable by the use of a suitable chiral auxiliary that is enantiomerically pure and able to transfer enantiomers into a diastereomeric environment. Three main classes of chiral auxiliaries for NMR spectroscopy have been developed to this purpose, among which chiral solvating agents (CSAs)^1−4^ stand out for their practicality of use, being simply mixed to the enantiomeric mixtures into the NMR tube without need for chemical derivatization and subsequent purification procedures. The majority of CSAs are simple chiral platforms, the functional groups of which may be modified to address the enantiodiscriminating efficiencies and versatilities. In some cases, rigid structures are privileged with a hydrogen-bond donor or acceptor and aromatic groups, the anisotropic effects of which are ultimately responsible for the chemical shift differentiation of the enantiomeric pairs. Design efforts are sometimes pursued to develop highly preorganized macrocyclic structures in favor of a pronounced enantiodiscriminating efficiency toward selected classes of chiral substrates, and more flexible structures could be preferred in view of enhancing the enantiodiscriminating versatility.

In spite of the vastness of structural features of CSAs, which makes them able to cover the chiral analysis of the majority of organic chiral compounds, the enantiomeric differentiation of amino acids remains a challenging task. As a matter of fact, only very few cases of the direct analysis of underivatized amino acids have been reported, such as in the case of the chiral crown ether reported by Wenzel^5^ or the flavonoid epigallocatechin gallate.^6^ Other CSAs offered interesting opportunities for the analysis of the *N*-tosyl^7−11^ and *N*-phthaloyl^12,13^ derivatives of amino acids, where the methyl group of the tosyl moiety also acts as the probe for the NMR enantiodifferentiation.

Therefore, leaving behind the ambitious purpose of obtaining CSAs for underivatized systems, the development of new versatile and efficient CSAs for the differentiation of amino acid derivatives remains a relevant topic, particularly when simple synthetic procedures are involved in the syntheses of both the CSA and the amino acid derivatives and, possibly, the moiety introduced in the amino acid affords itself suitable probe signals for the differentiation and quantification of the enantiomeric substrates in the NMR spectra.

**Figure 1:**
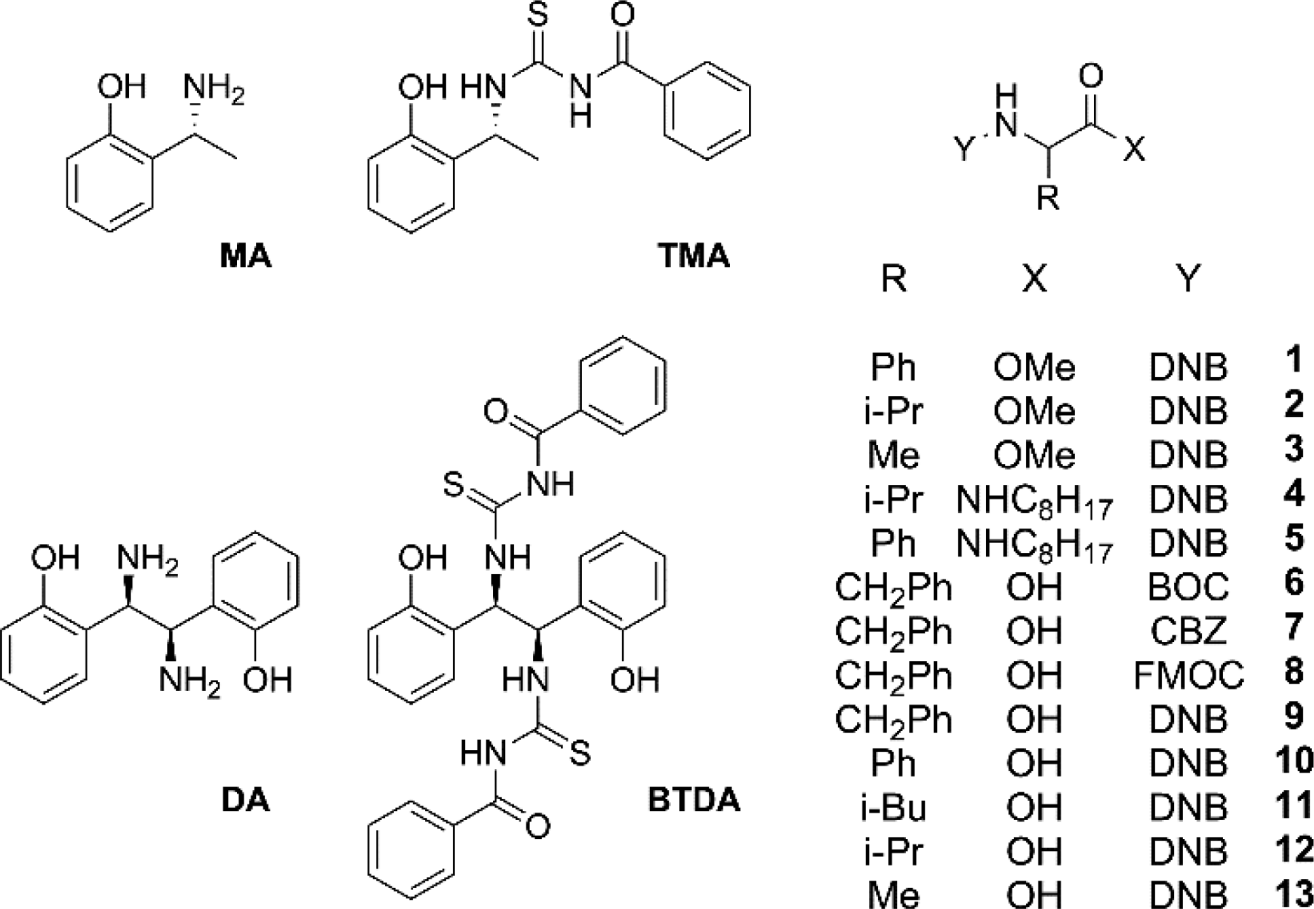
Amine precursors (**MA** and **DA**), their respective CSAs (**TMA** and **BTDA**), and derivatives **1**–**13** (DNB = 3,5-dinitrobenzoyl, BOC = *tert*-butyloxycarbonyl, CBZ = benzyloxycarbonyl, FMOC = fluorenylmethoxycarbonyl).

Treat potential of thiourea CSAs^13−25^ has been shown mainly in the past few years, and the **TMA** derivative (Figure 1) of commercially available 2-((1*R*)-1-aminoethyl)phenol (**MA**, Figure 1) has been recently proposed.^25^ Here, we explore the potentialities of a C_2_-symmetric chiral bis-thiourea derivative, **BTDA** (Figure 1), of commercially available (1*R*,2*R*)-1,2-bis(2-hydroxyphenyl)ethylenediamine (**DA**, Figure 1) as a chiral solvating agent for amino acid derivatives (Figure 1). **BTDA** is endowed with a tweezer-like structure that potentially has cooperative binding sites. Possible beneficial effects of a third achiral base additive (1,4-diazabicyclo[2.2.2]octane, DABCO, or *N*,*N*-dimethylpyridin-4-amine, DMAP) were also taken into consideration. ^1^H and ^13^C NMR enantiodiscrimination experiments were performed, and the chiral discrimination mechanism was carefully investigated by NMR.

## Results and Discussion

The reaction of **DA** with 2 equiv of benzoyl isothiocyanate proceeded selectively at the amino groups, leading to the thiourea derivative **BTDA** in a quantitative yield. No purification steps were required. **BTDA** showed a good solubility in CDCl_3_, which is privileged as a solvent in NMR enantiodiscrimination experiments.

The good solubility of both the CSA and the enantiomeric substrates in the same solvent constitutes a prerequisite for the optimization of enantiodiscrimination experiments to affect the stereoselective complexation equilibrium by changing the total concentration and either the CSA-to-substrate molar ratio or temperature. Therefore, different derivatizations of amino acids were searched to improve their CDCl_3_ solubility. Amino acids derivatized both at the amino and carboxyl functions showed very good solubilities in organic solvents.

In particular, amino acids with a 3,5-dinitrobenzoyl linked to the amino group offered a dual advantage. First, their 3,5-dintrophenyl protons resonate in a spectral region that is free of interference from the CSA signals and can therefore be used as basis for the detection and quantification of enantiomers. Importantly, the same aromatic moiety can favor π–π stacking interactions with aromatic groups of the CSA, thus stabilizing the diastereomeric solvates. The derivatization of the carboxyl group as a methyl ester (**1**–**3**) affords a sharp singlet, which is very useful for the quantification of enantiomers and also accurate when the magnitude of the nonequivalence is scarce. On the other hand, having an additional amide function at the carboxyl group, such as in **4** and **5**, could favor brush-type interactions with polyamide CSAs. In the case of amino acids with a free carboxylic group, the use of a base additive was needed as a solubilizing promoter, which in principle could play an active role in mediating the enantioselective interactions with the chiral auxiliary as was also demonstrated for the monomeric **TMA**.

### ^1^H NMR Enantiodiscrimination Experiments

Enantiodiscrimination experiments were carried out by adding one equivalent of **BTDA** to the CDCl_3_ solution of the selected amino acid derivative. Splittings of the NMR signals of enantiomeric substrates were detected and, for selected cases, the magnitude of the splittings (nonequivalence, i.e., the difference, in either hertz or parts per million, between the chemical shifts of the corresponding signals of the two enantiomers in the mixtures containing the CSA, ΔΔδ = |Δδ_R_ – Δδ_S_| = |δ_R_ – δ_S_|, where Δδ_R_ = δ_mixture_^R^ – δ_free_ and Δδ_S_ = δ_mixture_^S^ – δ_free_ are the complexation shifts) was compared to that obtained in the presence of the previously reported chiral auxiliary **TMA**.^25^

**Figure 2:**
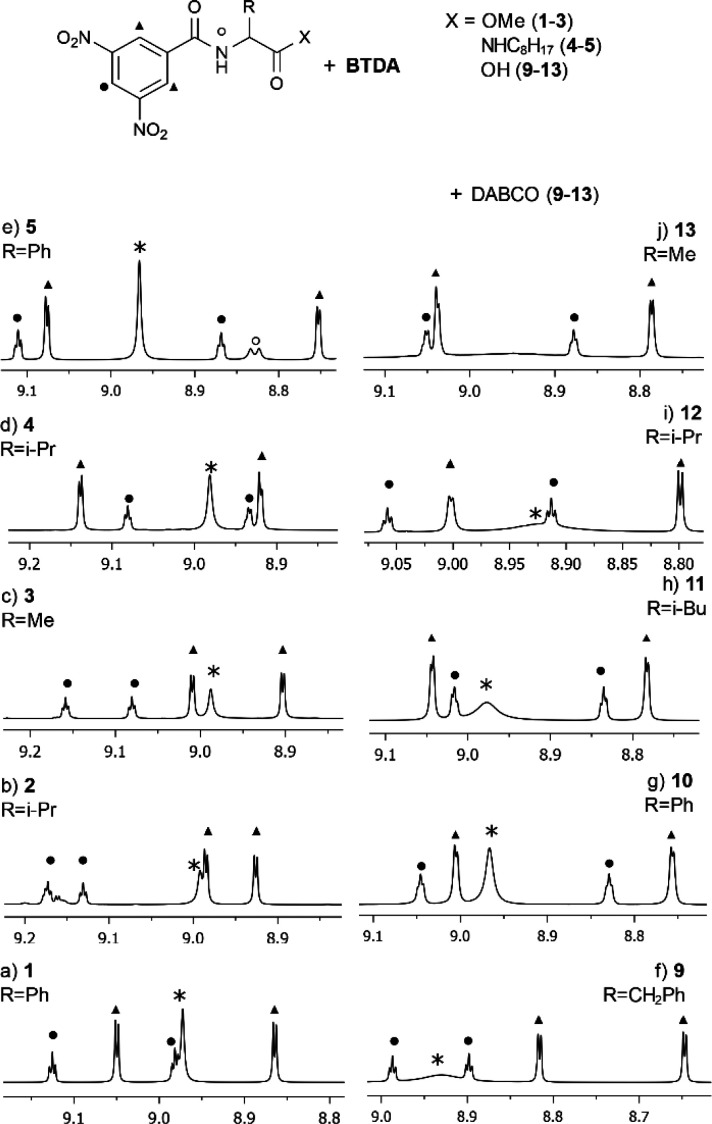
^1^H NMR (600 MHz, CDCl_3_, 25 °C) spectral regions corresponding to the *ortho-* and *para*-DNB protons of a 1:1 mixture of **BTDA** (30 mM)/racemic (a) **1**, (b) **2**, (c) **3**, (d) **4**, or (e) **5**. ^1^H NMR (600 MHz, CDCl_3_, 25 °C) spectral regions corresponding to the *ortho-* and *para*-DNB protons of a 1:1:1 mixture of **BTDA** (30 mM)/DABCO/racemic (f) **9**, (g) **10**, (h) **11**, (i) **12**, and (j) **13**. The asterisk (*) indicates the resonance of CSA.

Nonequivalences obtained for the derivatives **1**–**13** at 30 mM in the presence of 1 equiv of **BTDA** are collected in Table 1. NMR spectra corresponding to 3,5-dinitrophenyl, NH, and CH protons of **1**–**5** and **9**–**13** are shown in Figure 2 and Figures S1 and S2 in the Supporting Information, respectively.

**Table 1:** ^1^H NMR (600 MHz, CDCl_3_, 25 °C) Nonequivalences (ΔΔδ, ppm) of **1**–**5** (30 mM) in the Presence of an Equimolar Amount of **BTDA** and **6**–**13** (30 mM) in the Presence of Equimolar Amounts of **BTDA** and DABCO

substrate	**pDNB**^a^	**oDNB**^b^	**NH**	**CH**^c^	CO**-R**
**1**	0.141	0.186	0.210	0.043	0.036^d^
**2**	0.041	0.060	0.021	0.008	0.007^d^
**3**	0.078	0.106	0.009	0.005	0.008^d^
**4**	0.147	0.219	0.047	0.034	0.055^e^
**5**	0.243	0.324	0.113	0.028	0.160^e^
**6**^f^			0.089	0.030	
**7**			0.103	0.030	
**8**			0.147	0.021	
**9**	0.089	0.169	0.042	0.050	
**10**	0.216	0.247	0.173	0.049	
**10**^g^	0.209	0.256	0.074	0.042	
**11**	0.180	0.260	0.053	0.080	
**12**	0.145	0.202	0.045	0.096	
**13**	0.174	0.253	0.074	0.081	

a *para*-Proton of the DNB moiety.

b *ortho*-Protons of DNB moiety.

c Methine proton of the chiral center.

d R = O**Me**.

e R = **NH**C_8_H_17_.

f A nonequivalence of 0.031 ppm was measured on the methyl signal of BOC.

g In the presence of DMAP.

Regarding amino acid derivatives **1**–**5** with protected NH and COOH functions, **BTDA** induced very high nonequivalences for proton nuclei of the phenylglycine derivative **1**, which ranged from the minimum value of 0.036 ppm for the singlet of the methoxy group at the carboxyl function up to the very high value of 0.210 ppm for the NH proton. Lower values were measured in the analogous derivatives of valine (**2**) and alanine (**3**), but in any case the splittings at the 3,5-dinitrobenzoyl proton resonances guaranteed the accurate quantification of enantiomers. Improving hydrogen-bond donor and hydrogen-bond acceptor interactions, such as those for **4** and **5**, remarkably enhanced the differentiation of the 3,5-dinitrophenyl protons with nonequivalences, up to 0.243 and 0.324 ppm in the case of **5** for the *para*- and *ortho*-DNB, respectively. It is noteworthy that alkylamide derivatives **4** and **5** afforded a further NH signal, the nonequivalences of which were of 0.055 (**4**) and 0.160 ppm (**5**).

In the search for simplified derivatization procedures for the amino acids, several kinds of *N*-derivatizations of phenylalanine were attempted (**6**–**9**), leaving the carboxyl function underivatized. The above-mentioned derivatives were scarcely soluble in CDCl_3_, but their solubility could be improved by adding equimolar amounts of a strong organic base (DABCO or DMAP). Nonequivalences detected in the ternary mixtures **6**–**9**/**BTDA**/DABCO (1:1:1) were once again very high and scarcely affected by the nature of the derivatizing group linked to the amino group, since quite similar values were obtained for NH and CH protons (Table 1). While FMOC, BOC, and CBZ substrates **6**–**8** could be more relevant from a daily life perspective, the slow-exchanging *syn*- and *anti*-stereoisomeric forms could make the quantification of the enantiomers unreliable. In any case, nonequivalences spanning from 0.089 to 0.147 ppm were measured for the NH proton. Such interconverting species were not present in the case of **9**, which contained a 3,5-dinitrobenzoyl group at the nitrogen. Interestingly the enantiodiscrimination of BOC and CBZ derivatives of amino acids was reported by Tanaka et al.^12^ in the presence of substoichiometric amounts of a macrocyclic CSA.

NMR data collected for compounds **1**–**9** suggested favoring the analysis of *N*-3,5-dinitrobenzoyl derivatives of amino acids with free carboxyl functions in ternary mixtures CSA/substrate/base, as they had the best balance between the practicality of the synthesis of amino acid derivatives and the efficiency of the NMR enantiodiscrimination. Therefore, analogous derivatives of phenylglycine (**10**), leucine (**11**), valine (**12**), and alanine (**13**) were taken into consideration and analyzed in ternary mixtures containing equimolar amounts of DABCO and **BTDA**. In the case of **10**, nonequivalences of 0.247, 0.216, 0.173, and 0.049 ppm were measured for *ortho*- and *para*-protons of DNB and NH and the methine proton at the chiral center, respectively. Aromatic protons of the phenyl moiety were themselves remarkably perturbed, as demonstrated by 1D-ROESY and 1D-TOCSY experiments, allowing the extraction of their resonances. There was a nonequivalence of 0.170 ppm for the *ortho*-protons (Figure S3 in Supporting Information).

**Figure 3:**
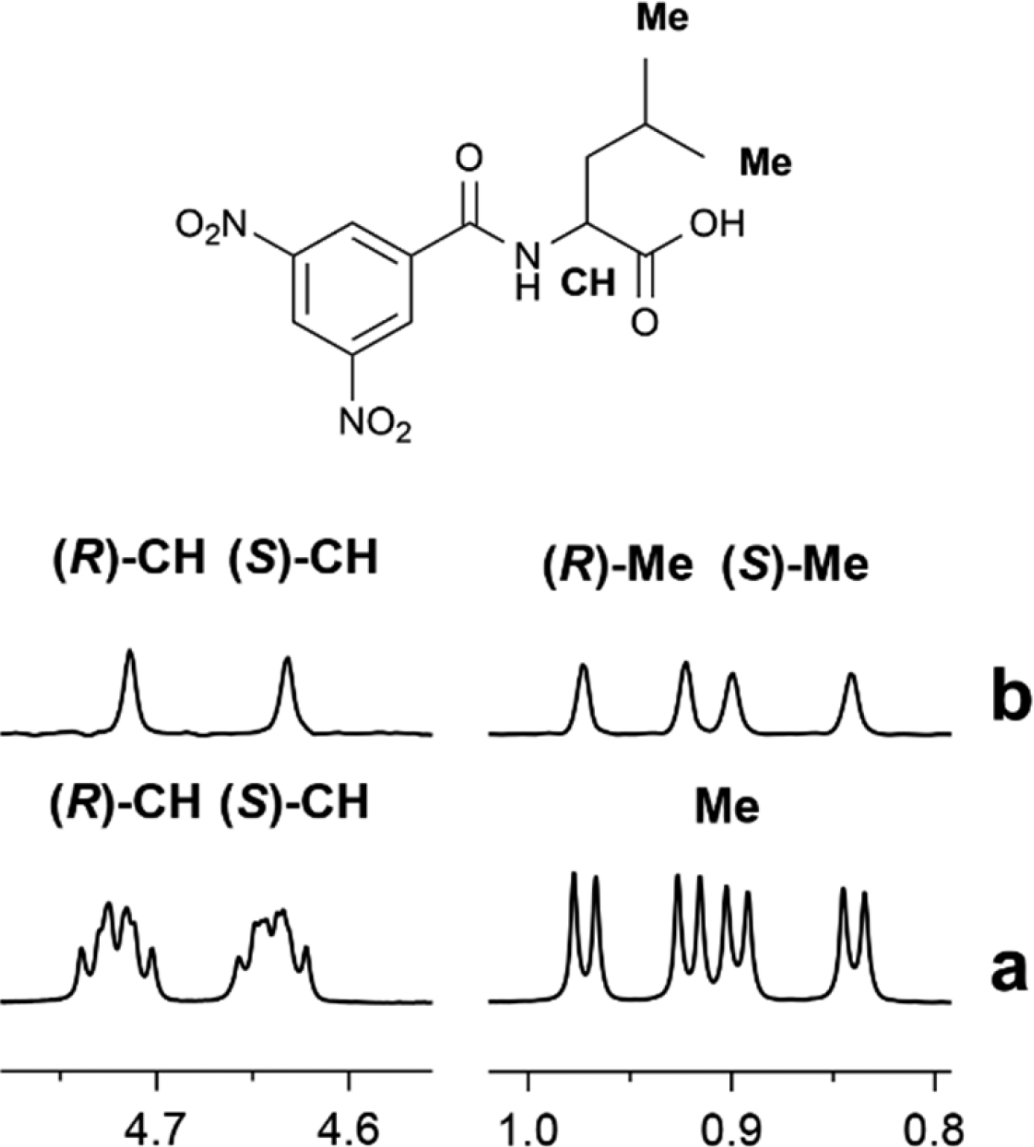
(a) ^1^H NMR (600 MHz, 30 mM, CDCl_3_, 25 °C) spectrum of the equimolar mixture **11**/**BTDA**/DABCO. (b) Pure-shift ^1^H NMR (600 MHz, 30 mM, CDCl_3_, 25 °C) spectrum of the equimolar mixture **11**/**BTDA**/DABCO.

Similarly, high nonequivalence values were also recorded for amino acid derivatives with branched aliphatic moieties, such as in the case of leucine **11** (Figure 3) and valine **12** in which remarkable doublings were induced on the diastereotopic methyl groups of the isopropyl moiety. Suppressing homonuclear scalar couplings by means of suitable pulse sequences allows an extension of the chiral analysis to complex or partially superimposed NMR signals, as shown in Figure 3. Singlets were obtained for the methyl and methine resonances of **11** by means of pure-shift experiments.

The high efficiency of **BTDA** was confirmed even in the case of the simplest amino acid derivative **13**, with the *ortho*- and *para*-protons of electron-poor ring differentiated by 0.253 and 0.174 ppm, respectively. Nonequivalences about of 0.080 ppm were obtained for the amide and methine protons. In any case, significantly higher nonequivalences were measured for *N*-3,5-dinitrobenzoyl derivatives with a free carboxylic function compared to those of the corresponding methyl ester compounds (**10** vs **1**, **12** vs **2**, and **13** vs **3**).

Interestingly, the high nonequivalences of almost every derivative were due to major complexation shifts of the (*R*)-enantiomer with respect to the (*S*)-enantiomer (Table S1 in Supporting Information). As the result of the interaction with the CSA, the *ortho*- and *para*-protons of the dinitrobenzoyl moiety of the (*R*)-enantiomer were more shielded than those of the (*S*)-enantiomer. Unexpectedly, an enhanced low-frequency complexation shift was also detected for the NH proton of the (*R*)-enantiomer, suggesting that complexation shifts were heavily affected by the anisotropic effects produced by the aromatic moieties of the CSA rather than by the reinforcement of hydrogen bond interactions, which should have produced a major deshielding for the enantiomer that formed the tighter diastereomeric pair with the CSA ((*R*)-enantiomer, vide infra). Only the methine protons underwent a deshielding effect upon complexation, which was more enhanced for the (*R*)-enantiomer.

Concerning the effect of the nature of the third achiral component in the ternary CSA/substrate/base mixture, DMAP was also tried in the same experimental conditions (CDCl_3_, 30 mM, **10**/**BTDA**/base 1:1:1 mixture). The nonequivalences measured for the *ortho*- and *para*-protons of the 3,5-dinitrobenzoyl moiety were very similar to those of DABCO, whereas a smaller value was measured for the amide proton (Table 1, Figure S4 in the Supporting Information). In any case, DABCO was preferred due to the fact that it produced one singlet at 2.78 ppm.

To understand the role of the base beyond its solubilizing properties, binary **10**/**BTDA** and ternary **10**/**BTDA**/DABCO mixtures were compared using a CDCl_3_/DMSO-d6 solvent mixture that contained the minimum amount of DMSO-d6 (40 μL in 640 μL of CDCl_3_) needed to solubilize **10**. As shown in Figure S5 in the Supporting Information, larger nonequivalences were attained in the mixture containing DABCO (nonequivalences of 0.003 and 0.017 ppm in the absence and in the presence of DABCO, respectively), confirming the active role of the base in the stabilization of diastereomeric solvates.

Comparing **BTDA** to previously reported monomeric **TMA** shone light on the eventual cooperative behavior of the two thiourea moieties of **BTDA** in the interaction with the enantiomeric substrates (Table 2). **TMA** produced significantly lower differentiations of **1**–**5** and **9**–**13** resonances than **BTDA** did in the same experimental conditions (equimolar amounts of CSA at 30 mM, Table 2). **BTDA** and **TMA** showed very different dependencies on the dilution. In the 30–5 mM range, the enantiodifferentiation of **10** and **11** underwent 35% and 80% reductions for DNB protons in the presence of **BTDA** and **TMA**, respectively (Tables 2 and 3). Therefore, the use of **BTDA** as a CSA seemed greatly advantageous compared to the corresponding monomeric CSA, including when the scarce dependence on concentration gradients was considered. The nonequivalences measured for **10** were still remarkable at 5 mM with doublings of 0.140, 0.159, 0.113, and 0.052 ppm for *ortho*-DNB, *para-*DNB, NH, and methine protons, respectively (Table 3).

**Table 2:** ^1^H NMR (600 MHz, CDCl_3_, 25 °C) Nonequivalences (ΔΔδ = |δ_R_ – δ_S_|, ppm) of **1**–**5** (30 mM) and **9**–**13**/DABCO (30 mM, 1:1) in the presence of 1 equiv of **BTDA** or **TMA**

	**BTDA**				**TMA**			
	pDNB^a^	oDNB^b^	NH	CH^c^	pDNB^a^	oDNB^b^	NH	CH^c^
**1**	0.141	0.186	0.210	0.043	0.045	0.071	0.076	0.003
**2**	0.041	0.060	0.021	0.008	0.021	0.028	0.036	0.006
**3**	0.078	0.106	0.009	0.005	0.036	0.049	n.d.^d^	
**4**	0.147	0.219	0.047	0.034	0.028	0.056		0.009
**5**	0.243	0.324	0.113	0.028	0.072	0.126	0.012	n.d.^d^
**9**	0.089	0.169	0.042	0.050	0.021	0.047	n.d.^d^	
**10**	0.216	0.247	0.173	0.049	0.079	0.129	0.010	0.034
**11**	0.180	0.260	0.053	0.080	0.076	0.123	0.014	n.d.^d^
**12**	0.145	0.202	0.045	0.096	0.061	0.087	0.030	0.003
**13**	0.174	0.253	0.074	0.081	0.090	0.124	0.079	0.035

a *para*-Proton of the DNB moiety.

b *ortho*-Protons of the DNB moiety.

c Methine proton of the chiral center.

d Not determined.

**Table 3:** ^1^H NMR (600 MHz, CDCl_3_, 25 °C) Nonequivalences (ΔΔδ = |δ_R_ – δ_S_|, ppm) of **10** or **11**/DABCO (1:1) in the presence of **BTDA** or **TMA** at Different Molar Ratios and Concentrations

		ΔΔδ of **10**		ΔΔδ of **11**		
[sub] (mM)	CSA/sub molar ratio	**BTDA**	**TMA**	**BTDA**	**TMA**	
15	1:1	0.185	0.058	0.167	0.054	pDNB^a^
		0.208	0.094	0.239	0.086	oDNB^b^
		0.146	0.024	0.044	0.014	NH
		0.055	0.024	0.075	0.001	CH^c^
15	2:1	0.247	0.096		0.082	pDNB^a^
		0.294	0.156		0.132	oDNB^b^
		0.172	0.045		0.025	NH
		0.060	0.039		0.004	CH^c^
5	1:1	0.140	0.016	0.139		pDNB^a^
		0.159	0.027	0.194		oDNB^b^
		0.113	0.014	0.032		NH
		0.052	-	0.060		CH^c^

a *para*-Proton of the DNB moiety.

b *ortho*-Protons of the DNB moiety.

c Methine proton of the chiral center.

d Not determined.

Then CSA/substrate/DABCO (substrate/DABCO 1:1, 15 mM) solutions containing 1 equiv of dimeric CSA **BTDA** or 2 equiv of monomeric **TMA** were compared (Table 3). Adding 2 equiv of **TMA** to the **10**/DABCO mixture increased the nonequivalences of the *ortho*- and *para*- protons of DNB moiety of **10** to 0.156 and 0.096 ppm, respectively (Table 3). Additionally, 1 equiv of **BTDA** at 15 mM caused larger nonequivalences, thus confirming the existence of cooperative effects involving the two thiourea chains of the dimeric structure of **BTDA** in the interaction with the substrate.

To go deeper into this aspect, complexation stoichiometries of the systems (*R*)-**10**/**BTDA**/DABCO and (*S*)-**10**/**BTDA**/DABCO were compared via Job’s method^26^ (Figure S6 in the Supporting Information). In both cases, a well-defined maximum at the 0.5 mole fraction, corresponding to a 1:1 stoichiometry, was found. It is noteworthy that additional independently interacting units of **BTDA** should have caused a 1:2 complexation stoichiometry.

### ^1^H NMR Enantiomeric Purity Determination

**Figure 4:**
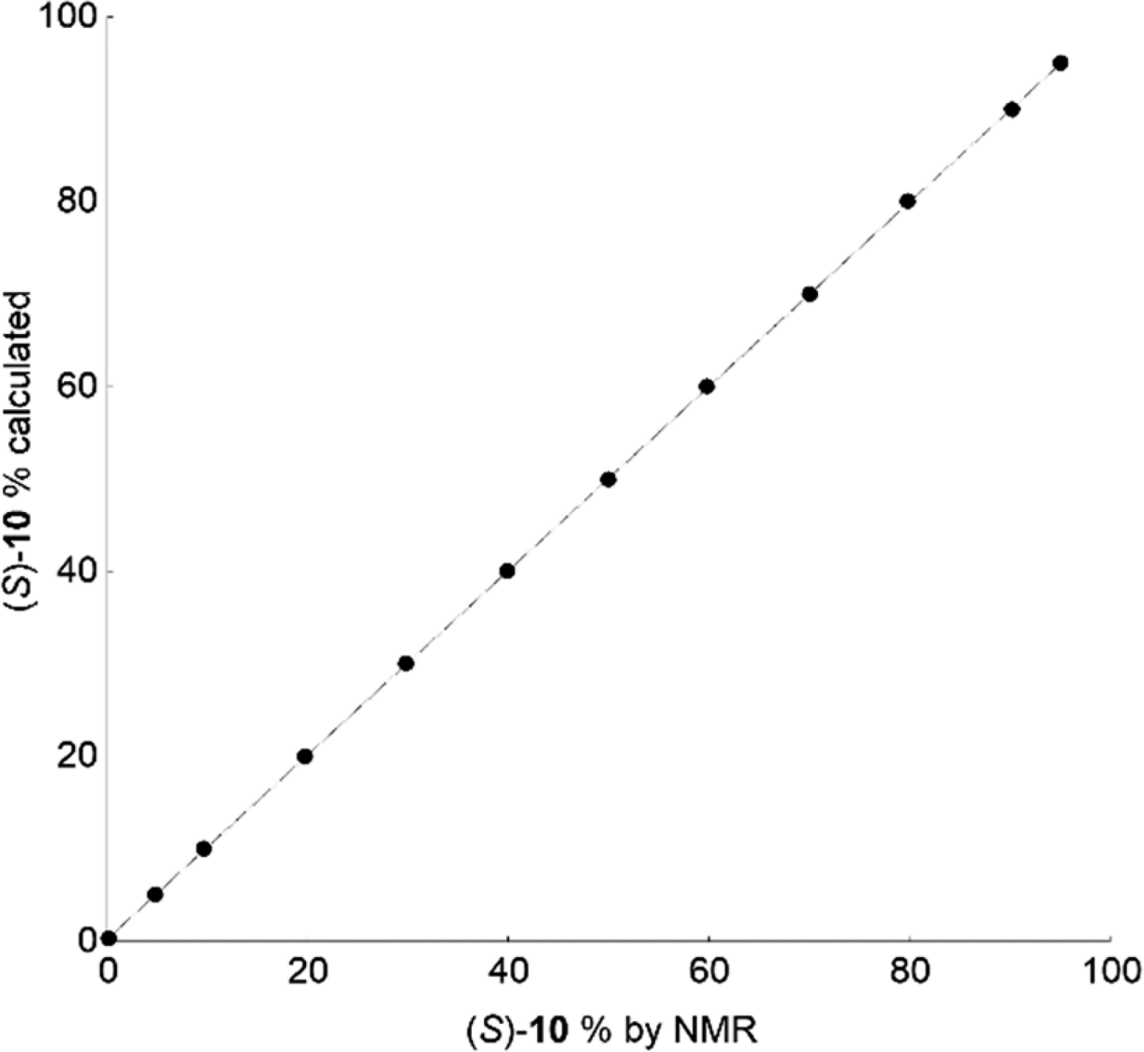
**BTDA** (30 mM)/DABCO (1:1) mixtures containing an equimolar amount of enantiomerically enriched **10** showing the correlation between the percentage of (*S*)-**10**, calculated from the mixed volumes of pure enantiomer stock solutions, and from that from the NMR integration of NH signals of **10**.

The bis-thiourea derivative **BTDA** was employed in the quantitative determination of the enantiomeric composition of enantiomerically enriched samples of **10** by integrating amide resonances of the two enantiomers in the ternary mixture **10**/**BTDA**/DABCO (1:1:1) at 30 mM. Values from the integration were in very good agreement with the values obtained on the basis of the knowledge of the volumes of stocks solutions employed for the preparation of NMR samples, as shown in Figure 4 (Tables S2). The good agreement was also confirmed in the analysis of mixtures with different contents of enantiomers for **9**–**13** (enantiomeric excess of 98%, Table S3 in the Supporting Information).

### ^13^C NMR Enantiodiscrimination Experiments

Usually, enantiodifferentiation experiments are performed by observing nuclei with a high sensitivity (^1^H, ^19^F, ^31^P, etc.), whereas low-sensitivity nuclei such as ^13^C and ^15^N are avoided. However, during the past decades^27−31^ technical advances have led to the reconsideration of observing such nuclei, which instead show great advantages in terms of spectral resolution in X{^1^H} decoupled spectra. As a matter of fact, in enantiodiscrimination experiments at the same signal-to-noise ratio, enantiomeric quantitative determinations by ^13^C{^1^H} NMR are more accurate than those by ^1^H NMR due to the lower line width of ^13^C{^1^H} NMR resonances.

**Figure 5:**
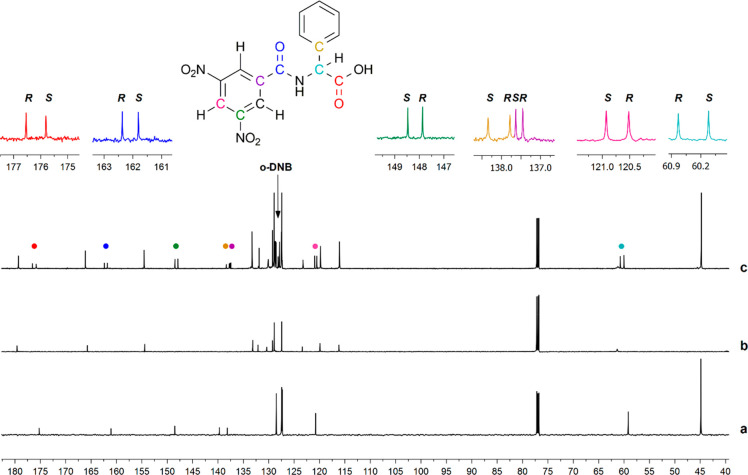
^13^C{^1^H} NMR spectra (150 MHz, 30 mM, 25 °C, CDCl_3_) of (a) the racemic **10**/DABCO equimolar mixture, (b) **BTDA**, and (c) the racemic **10**/**BTDA**/DABCO equimolar mixture.

To evaluate the possibility of using ^13^C nuclei observation in enantiodiscrimination experiments based on CSA **BTDA**, ^13^C{^1^H} NMR spectra of **10**/DABCO (1:1), **BTDA**, and **10**/**BTDA**/DABCO (1:1:1) were compared (Figure 5). ^13^C NMR signals of the ternary mixtures were characterized by means of the assignment of the protonated and quaternary carbons in 2D heteronuclear correlation experiments (HSQC and HMBC, Figures S7 and S8 in Supporting Information, respectively).

Remarkably high enantiodifferentiations were detected in the ternary mixture, and nonequivalence data are collected in Table 4. Nonequivalence values up to 0.7 ppm, the best separations, were detected for the carbonyl quaternary carbons of carboxyl and amide functions as well as for the C–NO_2_ carbons. Like in the case of **10**, remarkably high differentiations of ^13^C nuclei were detected in the ternary mixtures containing the other *N*-3,5-dinitrobenzoyl amino acid derivatives **11** and **13** with free carboxyl functions, with better nonequivalences for the carbonyl moieties, stereogenic carbon, *para*-DNB carbon, and quaternary carbons directly bound to the nitro groups (Table 4).

**Table 4:** ^13^C NMR (150 MHz, 30 mM, 25 °C, CDCl_3_) Nonequivalences of Derivatives **10**, **11**, and **13** in the Presence of Equimolar Amounts of **BTDA**/DABCO

	**10**	**11**	**13**
–**CO**–OH	0.73	0.20	0.08
–**C**–H	0.71	0.25	0.25
–**C**O–NH–	0.55	0.44	0.23
*ortho*-DNB	0.03	0.14	0.21
*para*-DNB	0.41	0.35	0.38
–**C**–NO_2_	0.59	0.55	0.53
**–**C–CH–	0.56	0.19	0.42
**–**C–CONH–	0.18	0.11	0.18

### Enantiodifferentiation Mechanism

To ascertain the molecular basis of the enantiodiscriminating efficiency of **10**, an accurate NMR investigation on the diastereomeric solvates (*R*)-**10**/**BTDA**/DABCO and (*S*)-**10**/**BTDA**/DABCO was carried out.

### CSA Conformation

**Figure 6:**
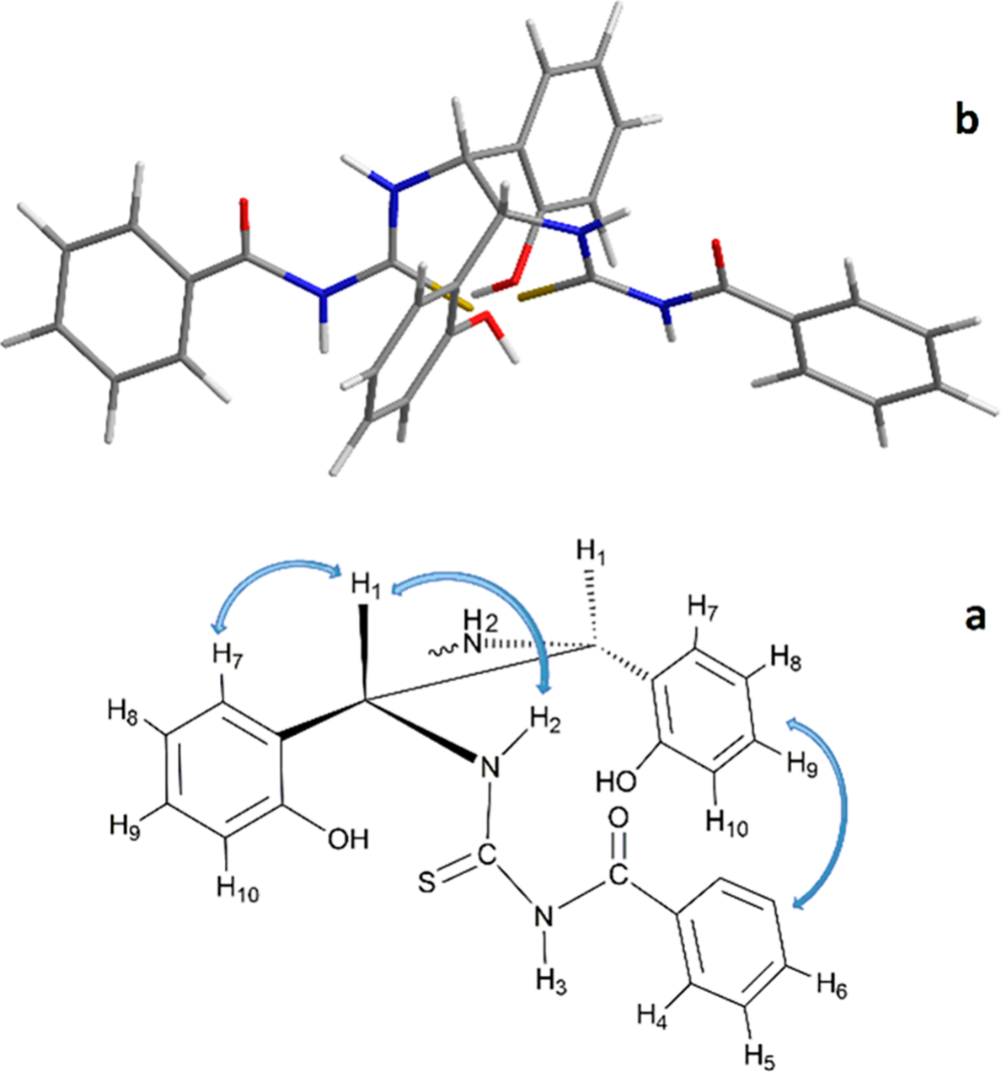
(a) Sawhorse projection of **BTDA** along the C(NH)–C(NH) bond in the cisoid arrangement and (b) 3D representation of **BTDA** according to NMR data. The blue arrows indicate ROE effects.

The conformation of pure CSA (30 mM) was defined by measuring through-space dipolar interactions by 1D and 2D ROE experiments. Due to the symmetry of the system, a unique set of signals was detected for both units that constitute dimeric CSA, which made the interpretation of the ROEs quite difficult. Interestingly, the inter-ROE H_7_–H_1_ was more intense with respect to the H_7_–H_8_ one (Figure S9b in Supporting Information), in accordance with the fact that the H_7_ proton of each phenolic moiety must lie in the proximity of the two cisoid C–H_1_ bonds in conformation A (depicted in Figure 6a). N–H_2_ protons belonging to the thiourea moiety (Figure S10 in Supporting Information) produced an intense ROE at the methine proton H_1_ frequency and a very weak dipolar interaction with H_3_ for which an intense ROE at the frequency of proton H_4_ of the proximal benzoyl moiety was found, which is in accordance with the conformation of the thiourea chain having cisoid and transoid N–H_2_/C–H_1_ and N–H_2_/N–H_3_ bonds, respectively, and an almost coplanar N–H_3_ and benzoyl moiety. Therefore, a cleft structure endowed with two major and minor grooves (Figure 6b) that are stabilized by an extended network of hydrogen-bond donor–hydrogen-bond acceptor interactions is supported by NMR data.

### **10/BTDA**/DABCO

**Figure 7:**
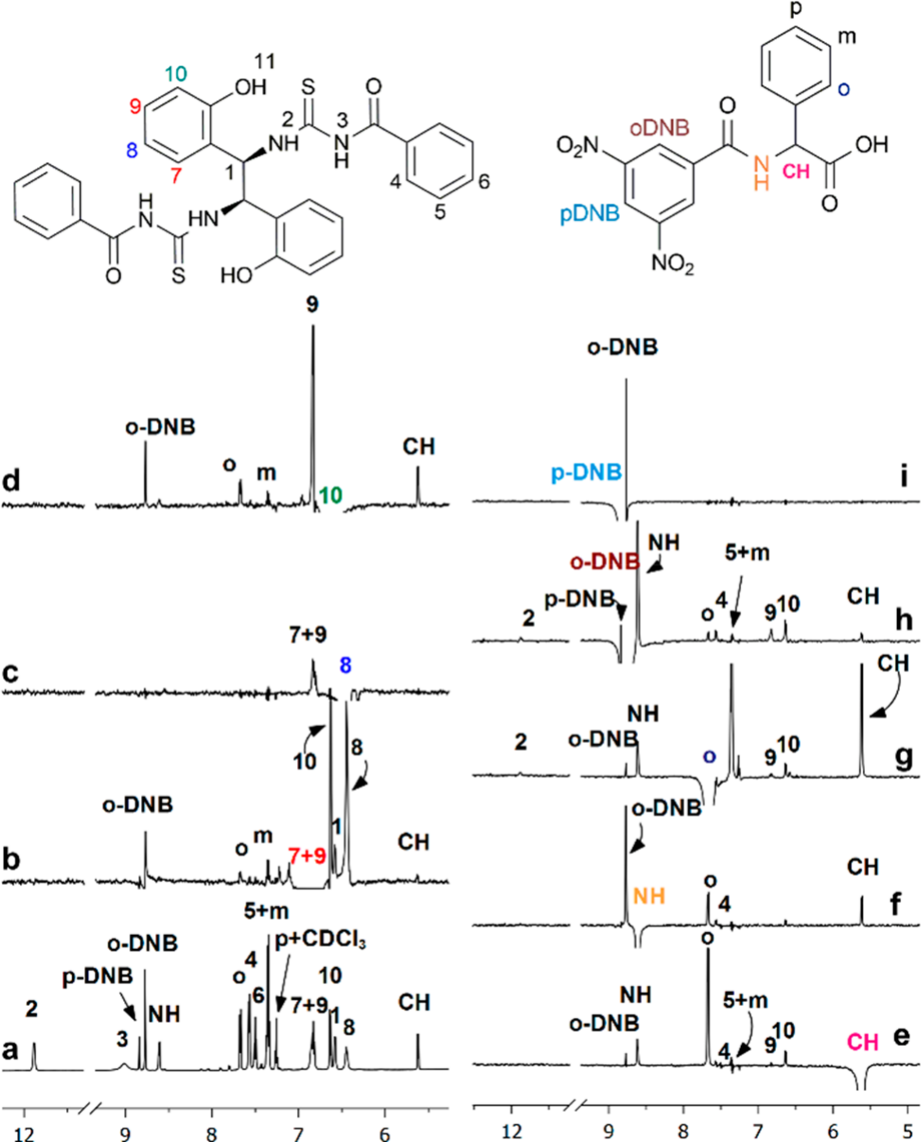
(a) ^1^H NMR (600 MHz, CDCl_3_, 30 mM, 25 °C) spectrum of the (*R*)-**10**/**BTDA**/DABCO equimolar mixture. 1D-ROESY experiments (mix 0.4 s) with the selective perturbation of (b) H_7_ + H_9_, (c) H_8_, (d) H_10_, (e) CH, (f) NH, (g) H_*o*_, (h) H_*o*-DNB_, and (i) H_*p*-DNB_ protons.

Regarding ternary mixtures **10**/**BTDA**/DABCO, ROE effects were detected between H_9_ and H_10_ (superimposed with H_7_) of **BTDA** and H_*o*-DNB_ of **10** (Figure 7b and d) in accordance with proximity of the electron-rich phenol of CSA and the electron-poor dinitrobenzoyl ring of **10**. Interestingly, any ROEs between H_*p*-DNB_ and phenolic protons were not detected (Figure 7f), favoring an edge-to-edge interaction rather than π–π stacking between the two aromatic moieties. Moreover, weak ROE effects between the protons H_*o*_ of the phenyl ring of **10** and H_10_ of the CSA were detected (Figure 7g). Dipolar interactions between phenolic protons of CSA and both phenyl and DNB groups of **10** can only coexist if the two groups are in the proximity of two distinct phenolic moieties of the CSA, with **10** bisecting the two major grooves originated by **BTDA**.

The CSA–substrate interaction was mediated by DABCO. As a matter of fact, DABCO protons produced relevant dipolar interactions (Figure S11 in Supporting Information) with amino acid protons, the π-acidic aromatic moiety of the CSA (benzoyl group), and its π-donor aromatic moiety (*o*-hydroxyphenyl moiety), which means that the base lies between all of these groups. This mechanism is also reasonable in consideration of the complexation stoichiometry. Due to the presence of the two nitrogens of DABCO, we could expect that 1 equiv of base binds 2 equiv of the amino acid derivative, giving rise at least to a certain population of 1:2 CSA/substrate complexes. However, we found a pure 1:1 complexation stoichiometry (Figure S6 in Supporting Information), which is in agreement with a favored interaction mechanism where one nitrogen of DABCO binds the carboxylate of the amino acid and the other one binds the acidic hydroxyl of the phenolic moiety. Interestingly, the intermolecular dipolar interaction with the amide proton of **10** was negligible, confirming that, as expected, the carboxyl function of **10** is involved in the interaction with the base.

**Figure 8:**
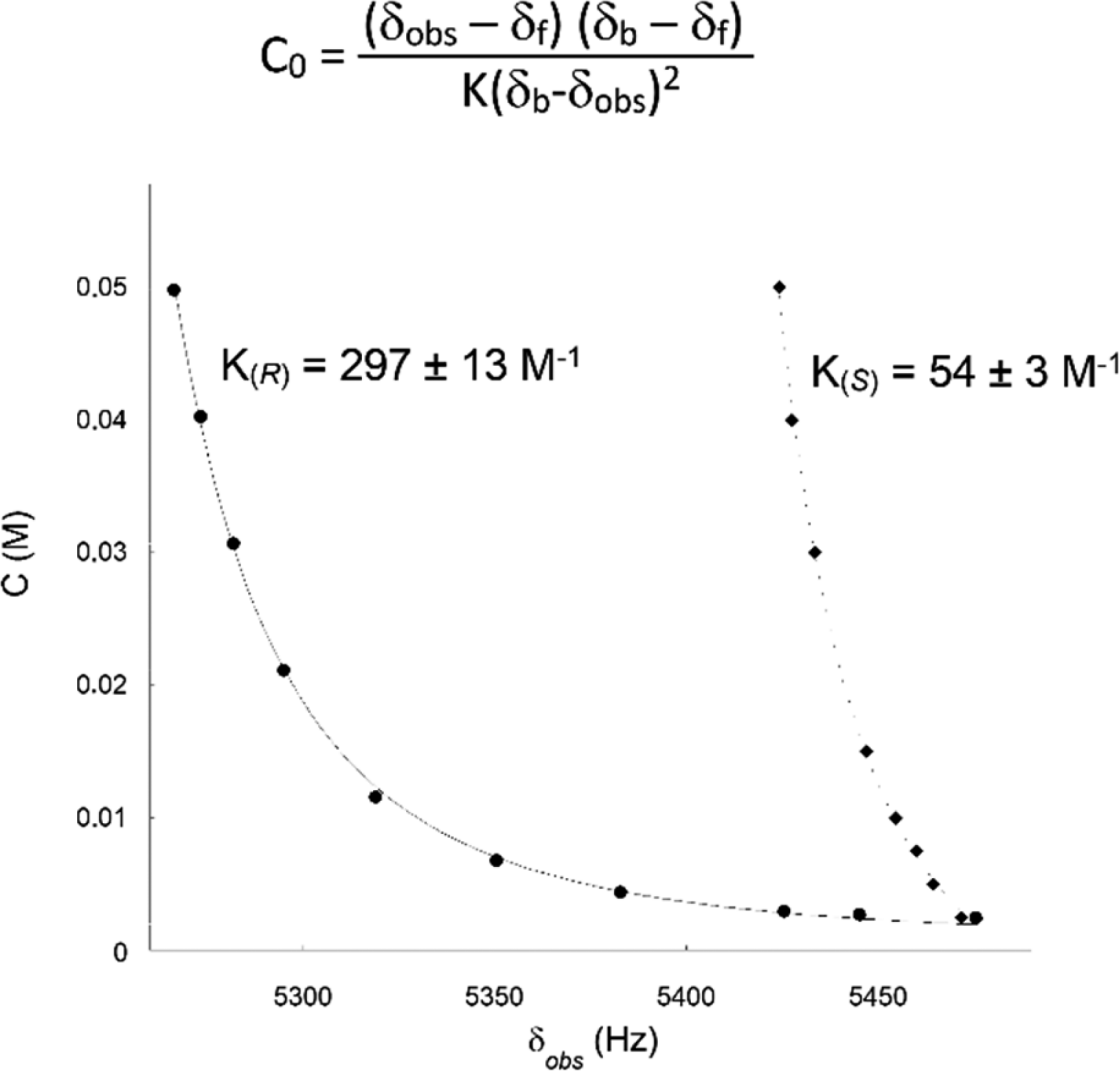
Nonlinear fitting of dilution data from the observed chemical shift of the *para*-DNB proton dependence on the concentration of **10** in equimolar mixtures of (*R*)-**10**/**BTDA**/DABCO (1:1:1) and (*S*)-**10**/**BTDA**/DABCO (1:1:1). δ_obs_, δ_b_, and δ_f_ in equation are the observed chemical shift and the chemical shift in the bound state and the free state, respectively.

Regarding CSA, a remarkably high ROE was detected between DABCO and the H_10_ phenolic proton adjacent to the OH group together with other non-negligible ROEs with protons of the benzoyl moiety of **BTDA**, in contrast to the negligible ROEs at the frequencies of NH protons of CSA. Therefore, it seems that DABCO is located at the central part of the cleft structure of the CSA, interacting mainly at its more acidic polar groups, i.e., phenolic hydroxyls, but simultaneously interacting with the carboxylate function of the enantiomeric substrates. In this way, the two enantiomers are both anchored to CSA through DABCO and bisect its major grooves. Interestingly, dipole–dipole interactions were qualitatively similar for both (*R*)- and (*S*)-enantiomers, but the interactions were weaker for the latter (Figures S11 and S12 in Supporting Information, respectively). Accordingly, the association constants, which calculated on the basis of the 1:1 stoichiometry using dilution data^32^ (Figure 8), were 297 ± 13 and 54 ± 3 M^–1^ for the (*R*)-**10/BTDA**/DABCO and (*S*)-**10/BTDA**/DABCO complexes, respectively.

## Conclusion

Nowadays, a major contribution to the design of new CSAs comes from receptors that are specifically oriented to increment the efficiency or versatility of enantiodiscrimination, avoiding complex derivatization procedures to analyze the enantiomeric substrates. Dimeric thiourea CSA **BTDA** is well-channelled in this trend, constituting a new tweezer-like artificial receptor endowed with a very high enantiodiscriminating ability toward *N*-3,5-dinitrobenzoyl derivatives of amino acids. The CSA is able to anchor the enantiomeric substrates through a complex network of hydrogen-bond donor and hydrogen-bond acceptor interactions and to cap them using a pool of aromatic moieties, which exert relevant anisotropic effects and hence cause sensitive changes of the chemical shifts. Among the aromatic moieties of the CSA, an especially relevant dual role is played by the phenolic units by virtue of the presence of an acidic hydroxy group and the electron-rich character of the aromatic ring, creating attractive interactions with the electron poor 3,5-dinitrophenyl group of derivatized enantiomers. The use of DABCO as a solubilizing additive makes it possible to employ the operatively simple derivatization of the sole amino group of the amino acid, leaving their carboxyl function underivatized. DABCO, beyond its role as solubilizing agent, actively assists the stabilization of the diastereomeric solvates by acting as a bridge, which enhances the hydrogen-bond donor character of the most acidic functional groups, i.e., the OH of CSA and the carboxyl function of the amino acid derivative. On this basis, the two enantiomers are bound by the CSA always preserving two kinds of attractive interactions that respectively occur at their carboxyl function and 3,5-dinitrophenyl moiety, the strengths of which are affected by the interchange of the two groups bound to the chiral center in the (*R*)-enantiomer with respect to the (*S*)-enantiomer. Hence, chiral discrimination is guaranteed.

## Experimental Section

### Materials

All commercially available substrates (**6**–**8**), reagents and solvents were purchased from Aldrich and used without further purification. Tetrahydrofuran (THF) was dried by distillation on potassium. Deuterated chloroform (CDCl_3_) used for the NMR analysis was acquired by Deutero GmbH. Derivatives **1**–**5** and **9**–**13** were prepared as described in refs (33) and (25), respectively. The NMR characterization is reported in ref (25).

### General Methods

^1^H and ^13^C{^1^H} NMR measurements were carried out on a spectrometer operating at 600 and 150 MHz for ^1^H and ^13^C nuclei, respectively. The samples were analyzed in a CDCl_3_ solution. ^1^H and ^13^C chemical shifts were referenced to tetramethylsilane (TMS) as the secondary reference standard, and the temperature was controlled (25 °C). For all the 2D NMR spectra, the spectral width used was the minimum required in both dimensions. The gCOSY (gradient correlation spectroscopy) and TOCSY (total correlation spectroscopy) maps were recorded using a relaxation delay of 1 s and 256 increments of four transients, each with 2*K*-points. For TOCSY maps, a mixing time of 80 ms was set. The 2D-ROESY (rotating-frame overhauser enhancement spectroscopy) maps were recorded using a relaxation time of 5 s and a mixing time of 0.4 s, and 256 increments of 16 transients of 2*K*-points each were collected. The 1D-ROESY spectra were recorded using a selective inversion pulse with transients ranging from 256 to 1024, a relaxation delay of 5 s, and a mixing time of 0.5 s. The gHSQC (gradient heteronuclear single quantum coherence) and gHMBC (gradient heteronuclear multiple bond correlation) spectra were recorded with a relaxation time of 1.2 s and 128–256 increments with 32 transients, each owith 2*K*-points. The gHMBC experiments were optimized for a long-range coupling constant of 8 Hz. ^1^H NMR and ^13^C{^1^H} NMR characterization data, reported below, refer to numbered protons and carbons from the chemical structures reported in Figures S13 and S14 in Supporting Information.

### Synthesis of **BTDA**

Benzoyl isothiocyanate (0.653 g, 4 mmol, 2 equiv) was added to **DA** (0.489 g, 2 mmol, 1 equiv) in CH_2_Cl_2_ (20 mL) at room temperature under a nitrogen atmosphere. The reaction mixture was stirred at room temperature for 24 h and monitored by ^1^H NMR. The solvent was removed by evaporation under vacuum to afford chemically pure **BTDA** in a nearly quantitative yield.

**BTDA**. White amorphous solid (1.126 g, 1.97 mmol, 98.7% yield), mp 164–166 °C. ^1^H NMR (CDCl_3_, 600 MHz): δ 11.99 (d, 1H, *J* = 2.5 Hz), 9.00 (s, 1H), 8.14 (br s, 1H), 7.77 (d, 2H, *J* = 7.6 Hz), 7.54 (t, 1H, *J* = 7.6 Hz), 7.42 (t, 2H, *J* = 7.6 Hz); 7.09 (d, 1H, *J* = 7.5 Hz), 6.98 (t, 1H, *J* = 7.5 Hz), 6.78 (d, 1H, *J* = 7.5 Hz), 6.72 (d, 1H, *J* = 2.5 Hz), 6.62 (t, 1H, *J* = 7.5 Hz). ^13^C{^1^H} NMR (CDCl_3_, 150 MHz): δ 179.6, 165.8, 154.5, 133.2, 132.1, 130.4, 129.3, 128.9, 127.5, 123.4, 119.9, 116.2, 61.4. Anal. Calcd for C_30_H_26_N_4_S_2_O_4_: C, 63.14; H, 4.59; N, 9.82. Found: C, 63.01; H, 4.57; N, 9.87.

## Supporting Information Available

Nonequivalences of NH and CH protons of amino acid derivatives, 1D ROESY and TOCSY spectra, ^1^H complexation shifts of amino acid derivatives, enantiodiscrimination of **10**, stoichiometry and enantiomeric purity determinations, 2D gHSQC maps, and ^1^H NMR spectra (PDF)
