# Supplementary material for: A Dimeric Thiourea CSA for the Enantiodiscrimination of Amino Acid Derivatives by NMR Spectroscopy
Source: J Org Chem. 2021 May 21;86(11):7381–9. doi: 10.1021/acs.joc.1c00340 (PMC8279476; doi:10.1021/acs.joc.1c00340)
Supplement: Supplementary file 1 — jo1c00340_si_001.pdf [file jo1c00340_si_001.pdf]

A dimeric thiourea CSA for the enantiodiscrimination of amino acid derivatives by NMR spectroscopy

Alessandra Recchimurzo,† Cosimo Micheletti,† Gloria Uccello Barretta and Federica Balzano*

Department of Chemistry and Industrial Chemistry, University of Pisa, via Moruzzi 13, 56124 Pisa, Italy.

†The authors contributed equally to this work.

*federica.balzano@unipi.it

Supporting Information

Table of Contents

Nonequivalences of NH and CH protons of amino acid derivatives in the presence of DABCO and **BTDA** (Figures S1-S2). S3

1D-ROESY and 1D-TOCSY spectra of (*S*)-**10**/**BTDA**/DABCO and (*R*)-**10**/**BTDA**/DABCO mixtures (Figure S3). S5

^{1}H complexation shifts of amino acid derivatives in the presence of DABCO and **BTDA** (Table S1). S5

Enantiodiscrimination of **10** in the presence of **BTDA** and DABCO or DMAP (^{1}H NMR spectra, Figure S4). S6

Enantiodiscrimination of **10** in the presence of **BTDA** or **BTDA**/DABCO (^{1}H NMR spectra, Figure S5). S6

Stoichiometry determination for (*R*)-**10**/**BTDA**/DABCO and (*S*)-**10**/**BTDA**/DABCO (Figure S6). S6

Enantiomeric purity determination (Table S2-S3). S7

2D gHSQC map of racemic **10**/**BTDA**/DABCO (Figure S7). S8

2D gHMBC map of racemic **10**/**BTDA**/DABCO (Figure S8). S8

1D ROESY spectra (Figures S9-S12). S9

^{1}H NMR (600 MHz, $CDCl_3$, 25 °C) and $^{13}C\{^1H\}$ NMR (150 MHz, $CDCl_3$, 25 °C) spectra of **BTDA** (Figures S13-S14). S11

^{1}H NMR (600 MHz, $CDCl_3$, 25 °C) spectrum of **1-5** (30 mM) in the presence of 1 equivalent of **BTDA** (Figures S15-S19) S13

^{1}H NMR (600 MHz, $CDCl_3$, 25 °C) spectrum of **9-13**/DABCO (30 mM, 1:1) in the presence of 1 equivalent of **BTDA** (Figures S20-S24) S14

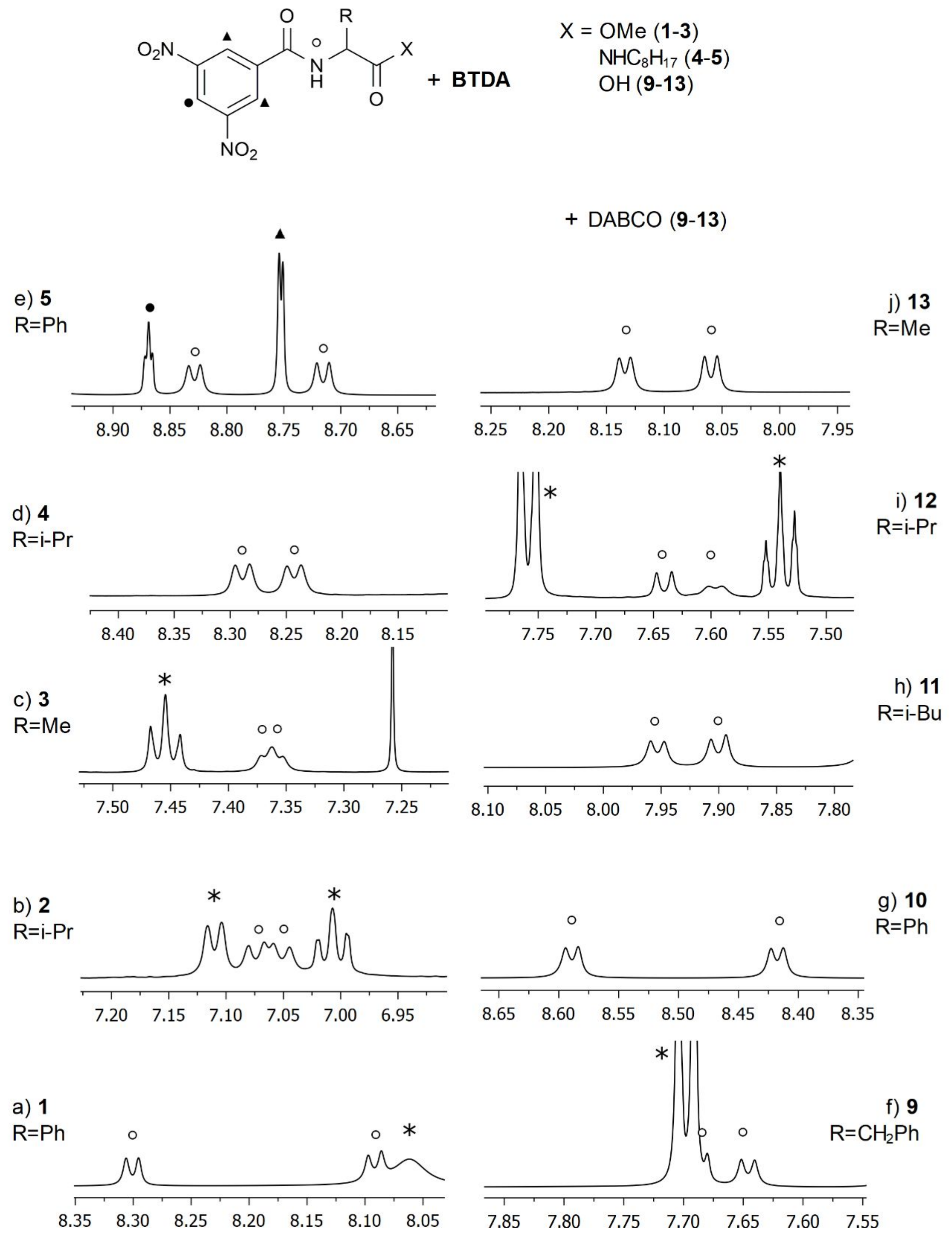

Figure S1. ^{1}H NMR (600 MHz, $CDCl_3$, 25 °C) spectral regions corresponding to NH protons of a 1-to-1 mixture of **BTDA** (30 mM)/racemic: a) **1**, b) **2**, c) **3**, d) **4**, e) **5**, and of 1-to-1-to-1 mixture **BTDA** (30 mM)/DABCO/racemic: f) **9**, g) **10**, h) **11**, i) **12**, j) **13**. *Resonance of CSA.

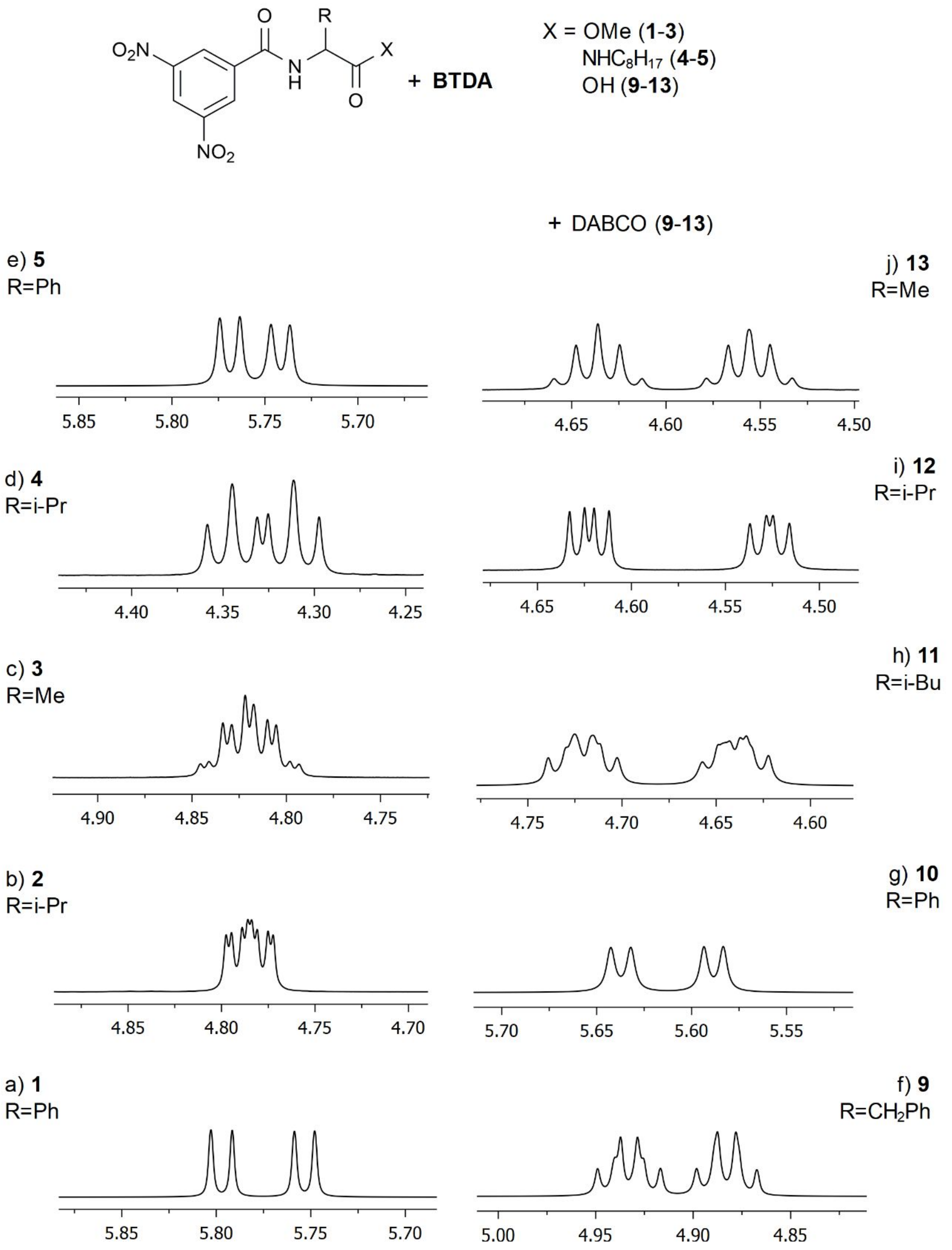

Figure S2. ^{1}H NMR (600 MHz, $CDCl_3$, 25 °C) spectral regions corresponding to methine protons of a 1-to-1 mixture of **BTDA** (30 mM)/racemic: a) **1**, b) **2**, c) **3**, d) **4**, e) **5**, and of 1-to-1-to-1 mixture **BTDA** (30 mM)/DABCO/racemic: f) **9**, g) **10**, h) **11**, i) **12**, j) **13**.

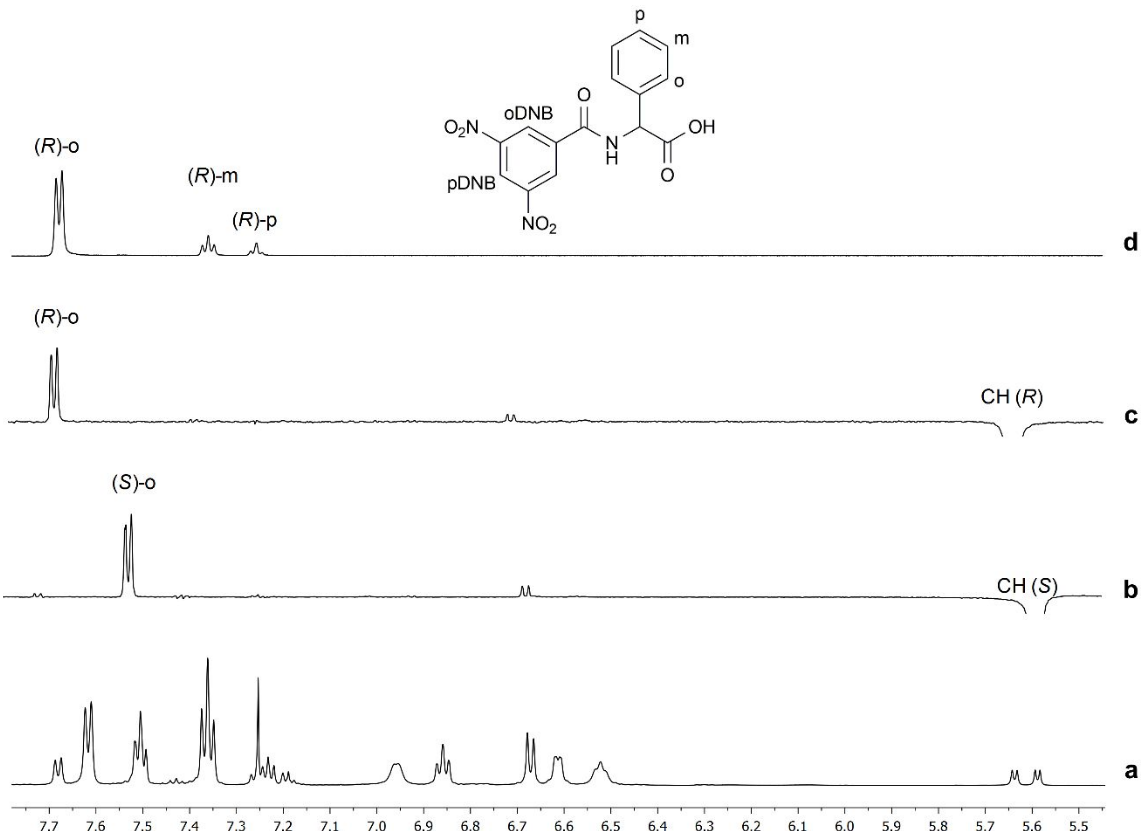

Figure S3. ^{1}H NMR (600 MHz, $CDCl_3$, 30 mM, 25 °C) spectral regions of **10**/**BTDA**/DABCO equimolar mixture (a); 1D-ROESY experiments (mix 0.4 s) with selective perturbation of CH in (*S*)-**10**/**BTDA**/DABCO (b) and (*R*)-**10**/**BTDA**/DABCO (c) equimolar mixtures; 1D-TOCSY experiment of ortho protons of substrate in (*R*)-**10**/**BTDA**/DABCO equimolar mixture (d).

Table S1. Complexation shifts (600 MHz, $CDCl_3$, 25 °C, $\Delta\delta = \delta_{mixture} - \delta_{free}$, ppm) of **9**-**13** (30 mM) in the presence of 1 equivalent of DABCO and **BTDA**.

Substrate	Δδ (ppm)			
	pDNB[a]	oDNB[b]	CH[c]	NH
**9**	-0.181 (R)<br>-0.092 (S)	-0.204 (R)<br>-0.035 (S)	+0.147 (R)<br>+0.097 (S)	-0.057 (R)<br>-0.015 (S)
**10**	-0.283 (R)<br>-0.067 (S)	-0.255 (R)<br>-0.008 (S)	+0.174 (R)<br>+0.125 (S)	+0.230 (R)<br>+0.057 (S)
**11**	-0.254 (R)<br>-0.074 (S)	-0.198 (R)<br>+0.062 (S)	+0.092 (R)<br>+0.012 (S)	-0.089 (R)<br>-0.036 (S)
**12**	-0.206 (R)<br>-0.061 (S)	-0.195 (R)<br>+0.007 (S)	+0.090 (R)<br>-0.006 (S)	+0.092 (R)<br>+0.047 (S)
**13**	-0.234 (R)<br>-0.060 (S)	-0.198 (R)<br>+0.055 (S)	+0.154 (R)<br>+0.073 (S)	+0.195 (R)<br>+0.269 (S)

[a]Para proton of DNB moiety. [b]Ortho protons of DNB moiety. [c]Methine proton of chiral center.

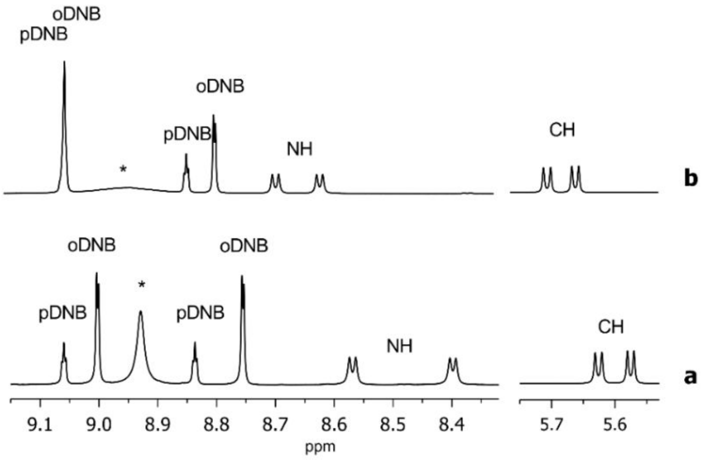

Figure S4. ^{1}H NMR (600 MHz, $CDCl_3$, 25 °C) spectral regions corresponding to the *ortho-* and *para*-DNB, NH and methine protons of **10** (30 mM) in 1:1:1 mixture: a) **10**/**BTDA**/DABCO, b) **10**/**BTDA**/DMAP. *indicates CSA signals.

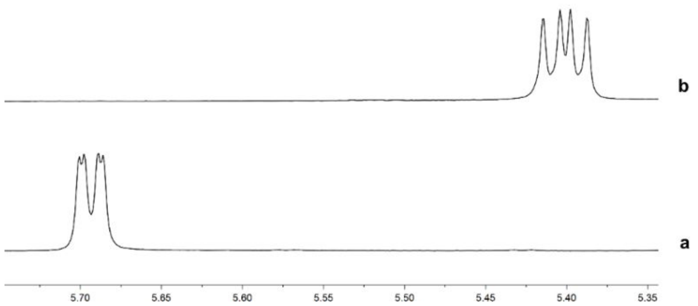

Figure S5. ^{1}H NMR (600 MHz, $CDCl_3$/DMSO-d6, 25 °C) spectral regions corresponding to CH proton of **10** (30 mM) in **BTDA/10** (a) and in **BTDA**/**10**/DABCO (b) mixtures (1:1:1).

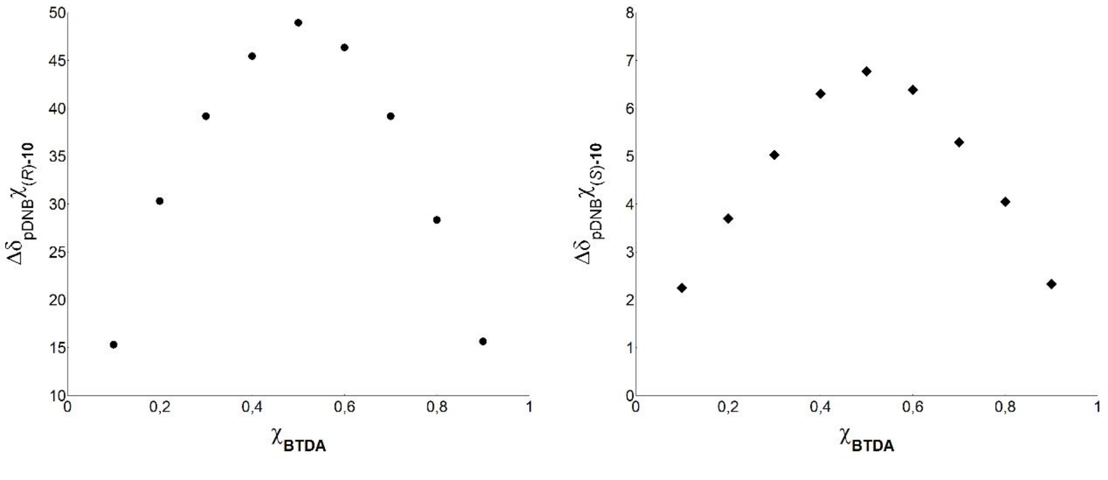

Figure S6. Stoichiometry determination based on *para* protons of DNB group for (*R*)-**10**/**BTDA**/DABCO (●) and (*S*)-**10**/**BTDA**/DABCO (◆)complexes.

Table S2. Comparison between the data of actual enantiomeric excess (ee %) of (*S*)-**10**, calculated from the mixed volumes of pure enantiomer stock solutions, and ee (%) of (*S*)-**10** determined by integration of the ^{1}H-NMR signals relating to the NH proton of **10** in **10**/**BTDA**/DABCO mixtures

Actual ee (%)	ee by NMR (%)
90	90.26
80	80.52
60	59.72
40	40.26
20	19.76
0	0.12
-20	-20.14
-40	-40.22
-60	-60.46
-80	-80.58
-90	-90.24
-99.4	-99.50

Table S3. Enantiomeric excess (ee %) determined by ^{1}H-NMR integration of signals for substrates **9**-**13** (30 mM, R:S=99:1, ee 98%) in the presence of **BTDA**/DABCO (30 mM, 1:1)

	ee by NMR (%)
**9**	-97.70
**10**	-98.22
**11**	-97.86
**12**	-98.18
**13**	-98.26

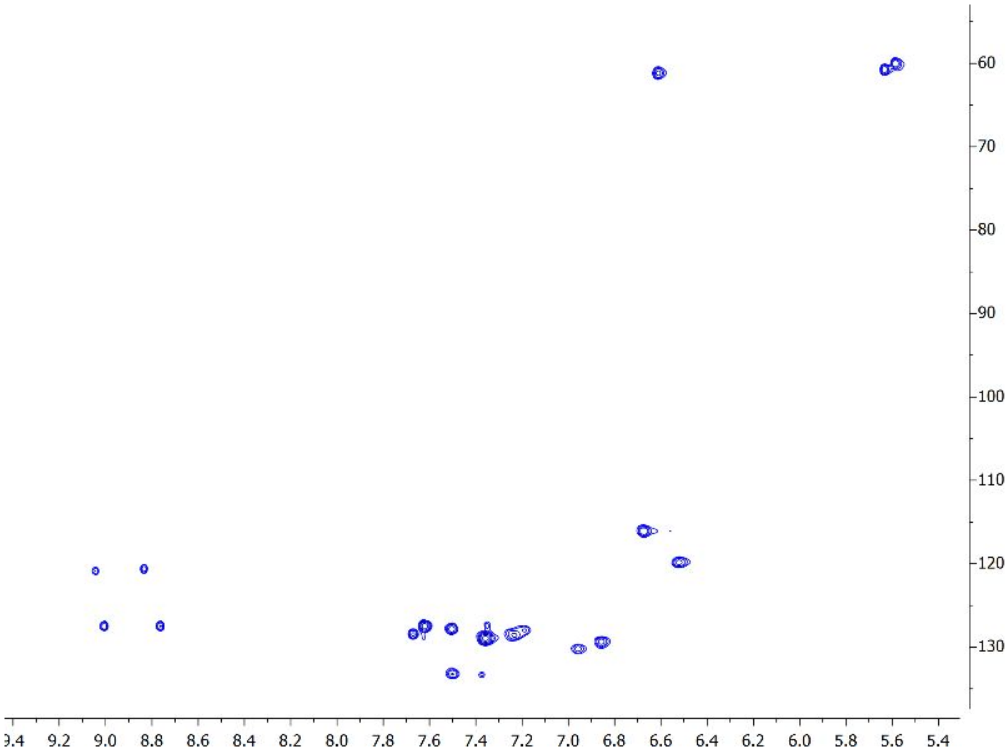

Figure S7. 2D gHSQC map (600 MHz, 30 mM, 25 °C, $CDCl_3$) of equimolar mixture of racemic **10**/**BTDA**/DABCO.

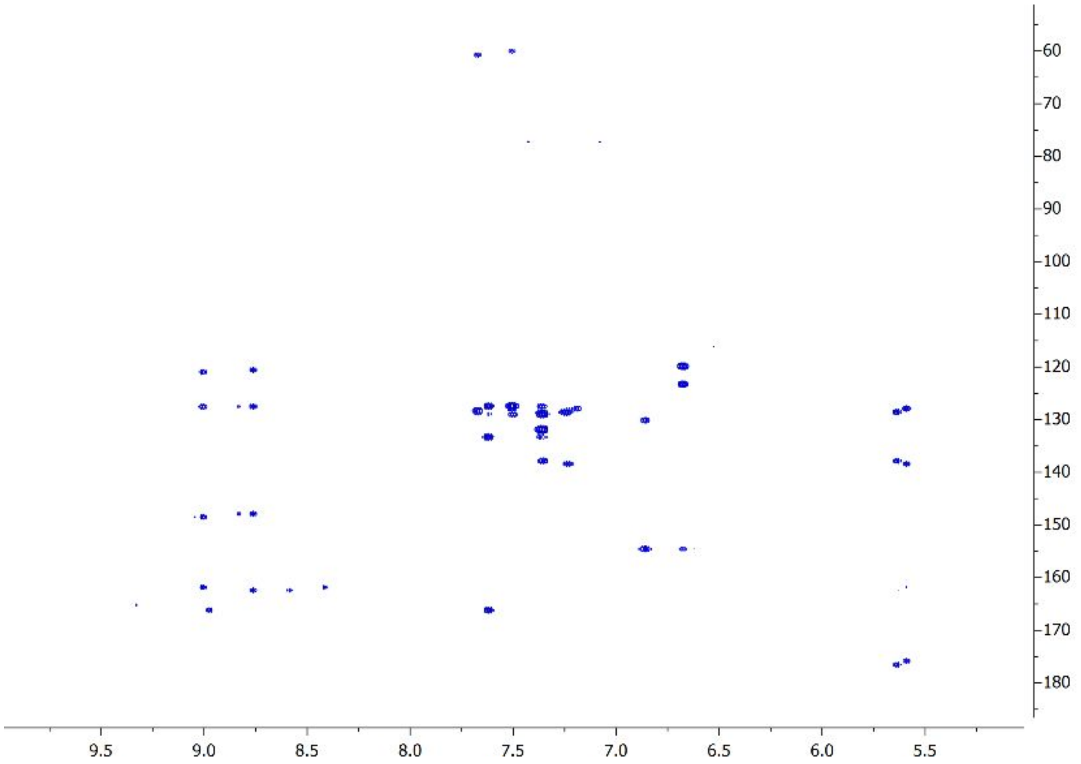

Figure S8. 2D gHMBC map (600 MHz, 30 mM, 25 °C, $CDCl_3$) of equimolar mixture of racemic **10**/**BTDA**/DABCO.

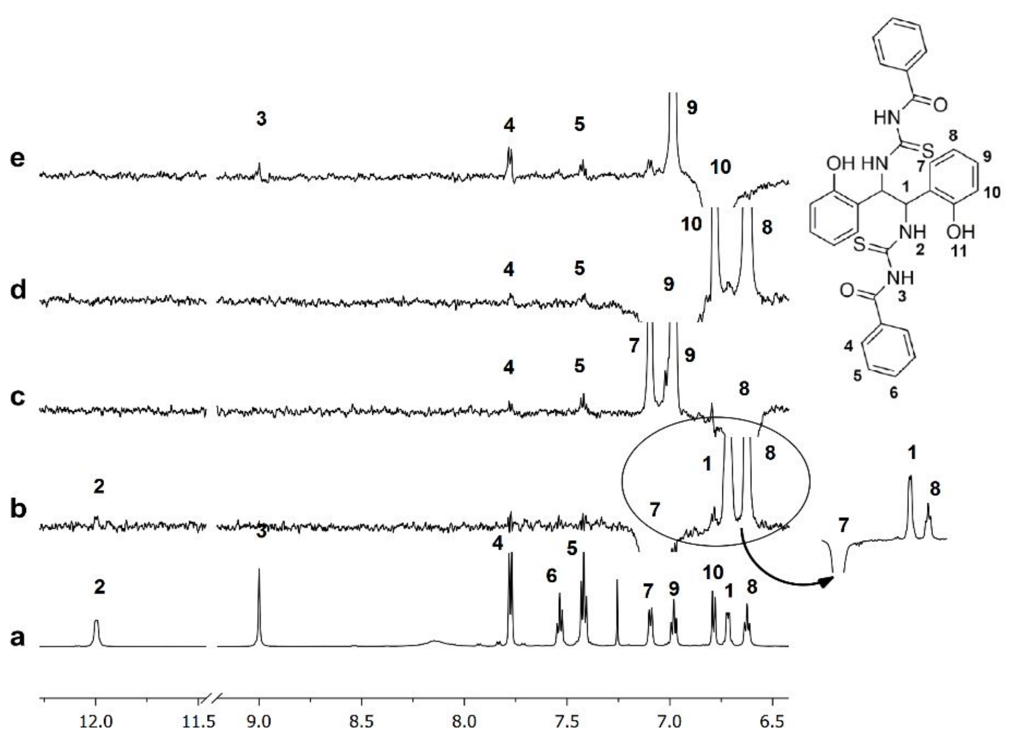

Figure S9. ^{1}H NMR spectrum (600 MHz, 30 mM, $CDCl_3$, 25 °C) of **BTDA** (a) and 1D-ROESY experiments (mix 0.4 s) with selective perturbation of H_7 (b), H_8 (c), H_9 (d), and H_{10} (e) protons.

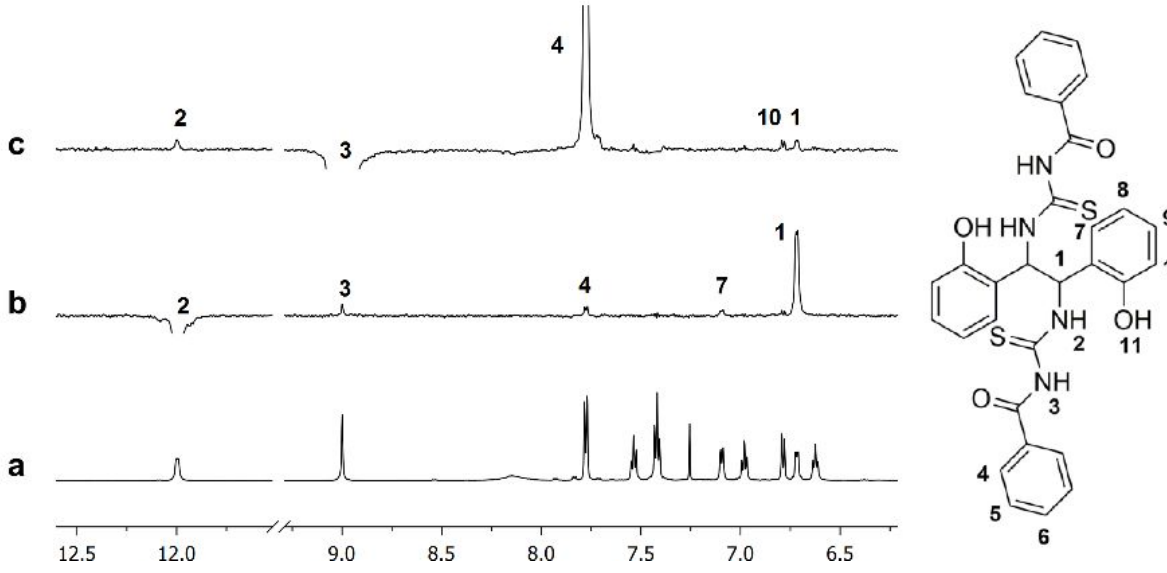

Figure S10. ^{1}H-NMR spectrum (600 MHz, 30 mM, $CDCl_3$, 25 °C) of **BTDA** (a) and 1D-ROESY experiments (mix 0.4 s) with selective perturbation of N-H_2 (b) and N-H_3 (c).

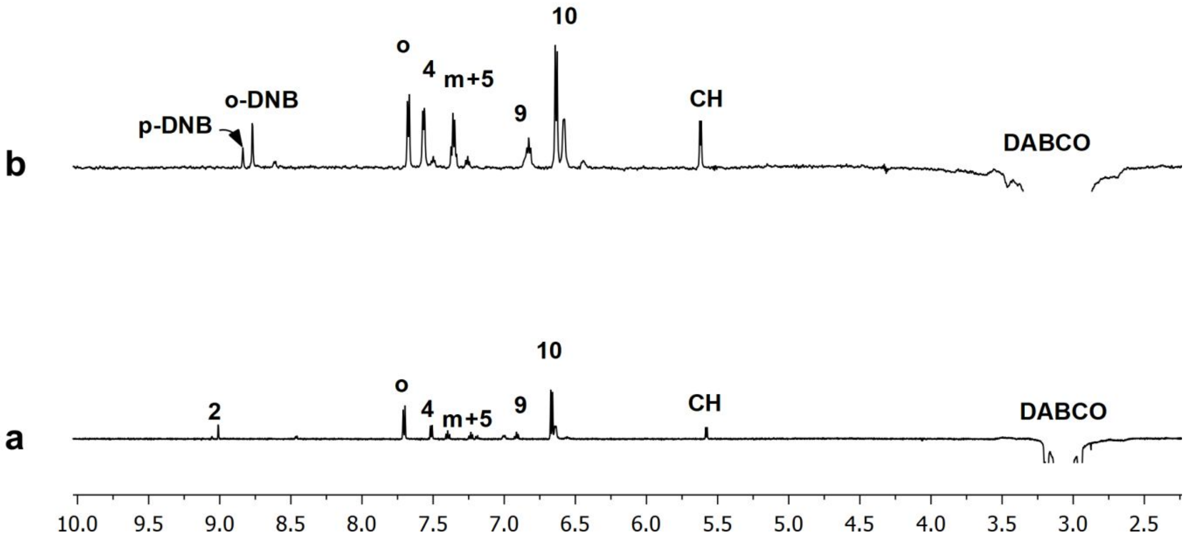

Figure S11. 1D-ROESY experiments (600 MHz, 30 mM, $CDCl_3$, 25 °C, mix 0.4 s) with selective perturbation of DABCO protons in (*S*)-**10**/**BTDA**/DABCO (a) and (*R*)-**10**/**BTDA**/DABCO (b) mixtures (1:1:1).

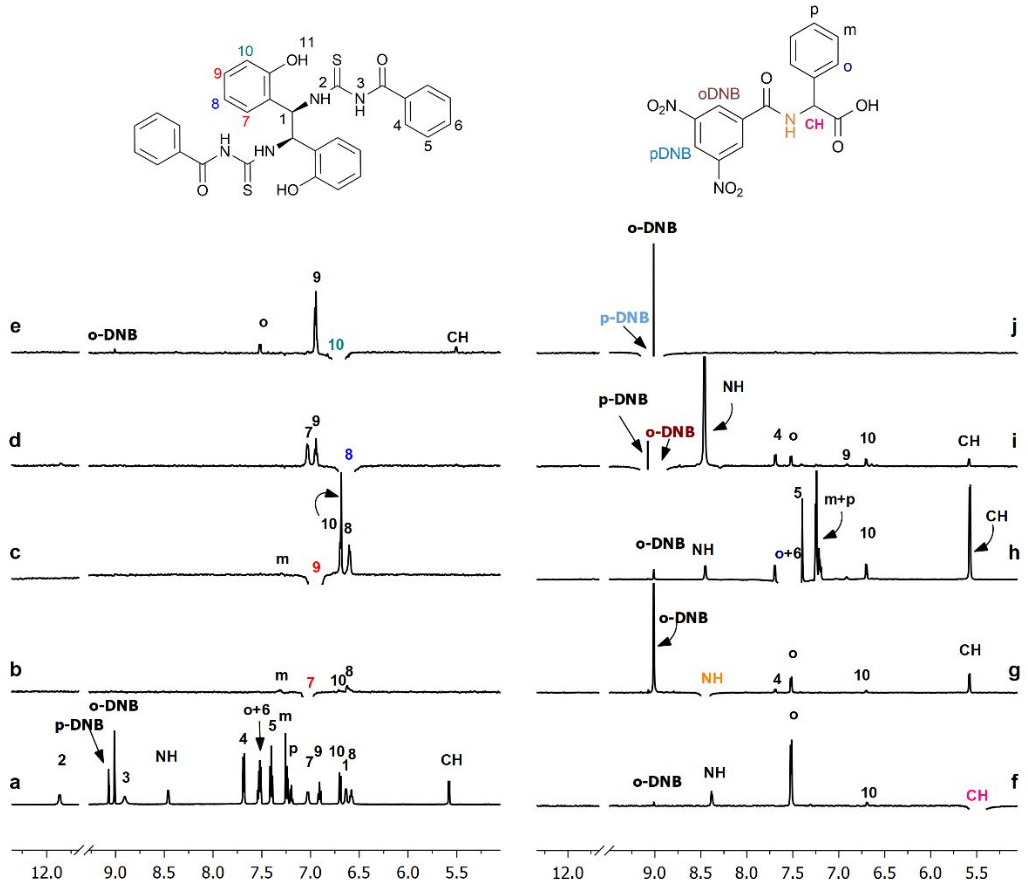

Figure S12. ^{1}H-NMR (600 MHz, $CDCl_3$, 30 mM, 25 °C) spectrum of (*S*)-**10**/**BTDA**/DABCO equimolar mixture (a) and 1D-ROESY experiments (mix 0.4 s) with selective perturbation of H_7 (b), H_9 (c), H_8 (d) H_{10} (e), CH (f), NH (g), H_o (h), $H_{o\text{-DNB}}$ (i), $H_{p\text{-DNB}}$ (j) protons.

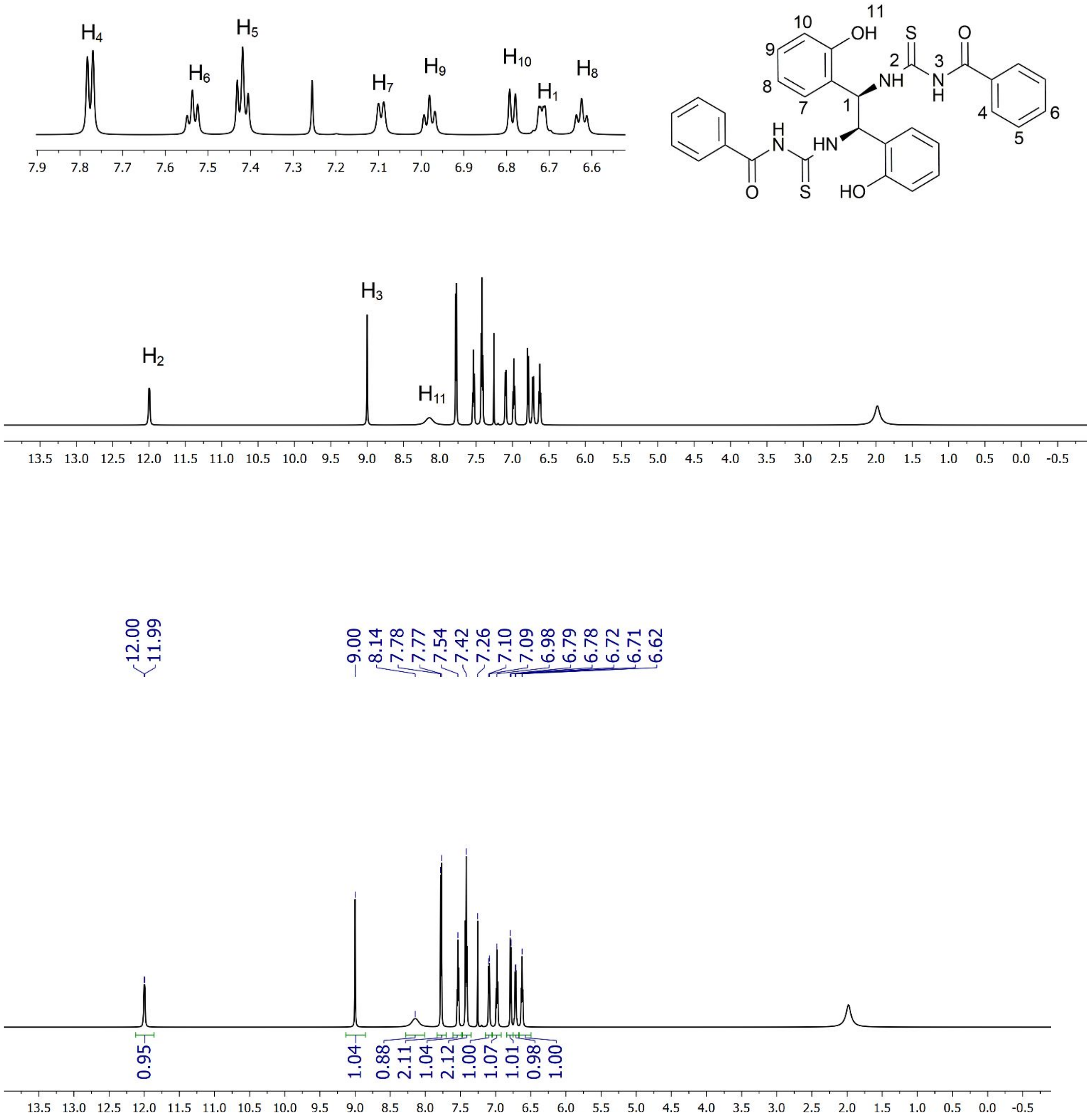

Figure S13. ^{1}H NMR (600 MHz, $CDCl_3$, 25 °C) spectrum of **BTDA**.

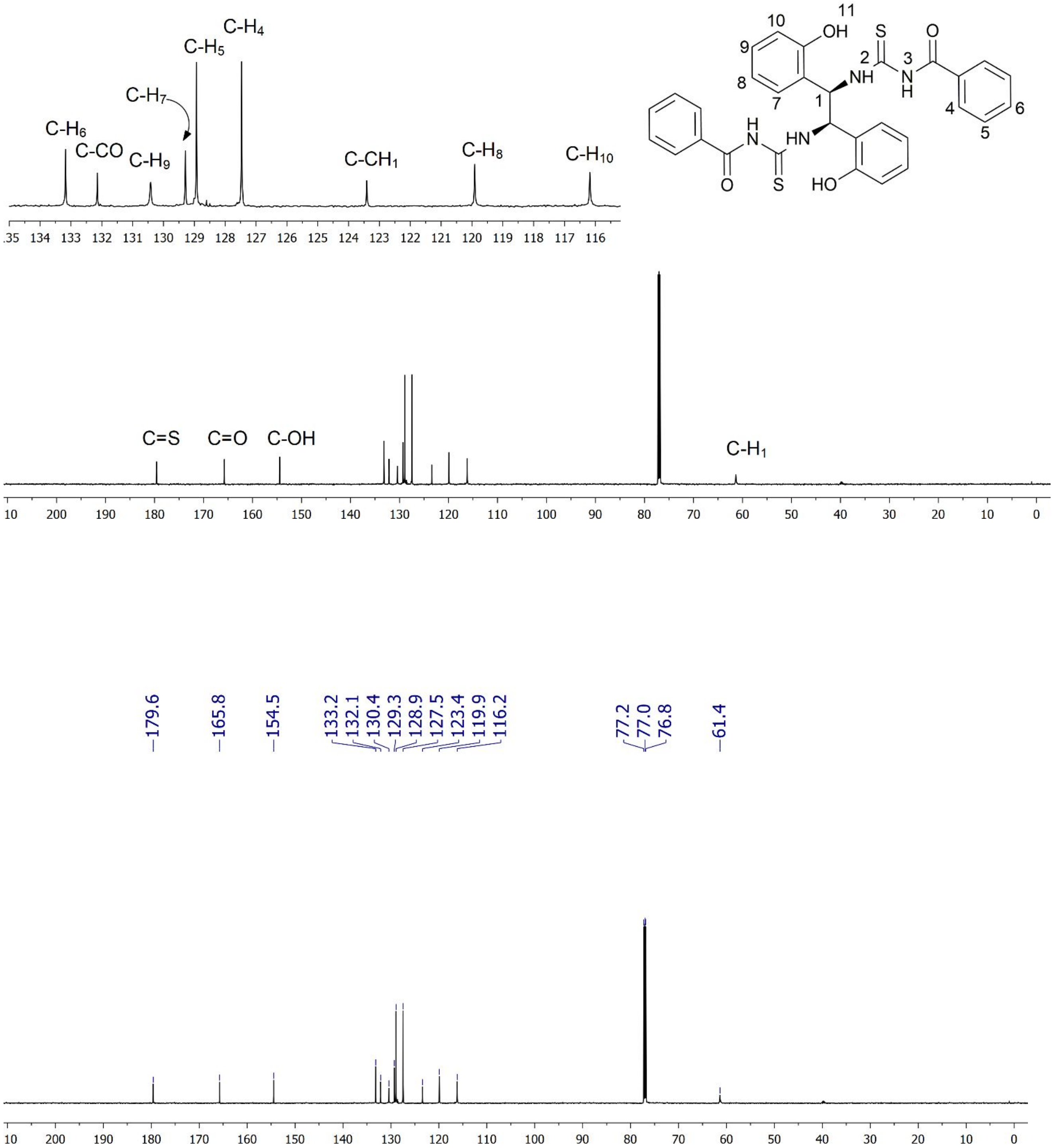

Figure S14. $^{13}C\{^1H\}$ NMR (150 MHz, $CDCl_3$, 25 °C) spectrum of **BTDA**.

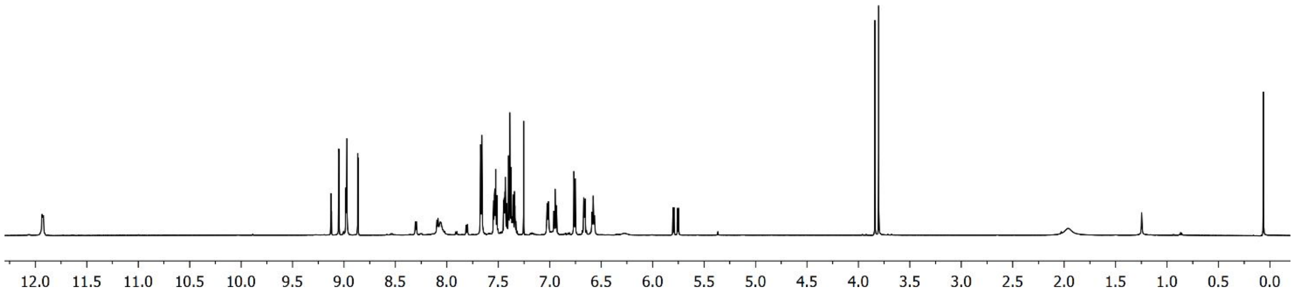

Figure S15. ^{1}H NMR (600 MHz, $CDCl_3$, 25 °C) spectrum of equimolar mixture **1**/**BTDA** (30 mM).

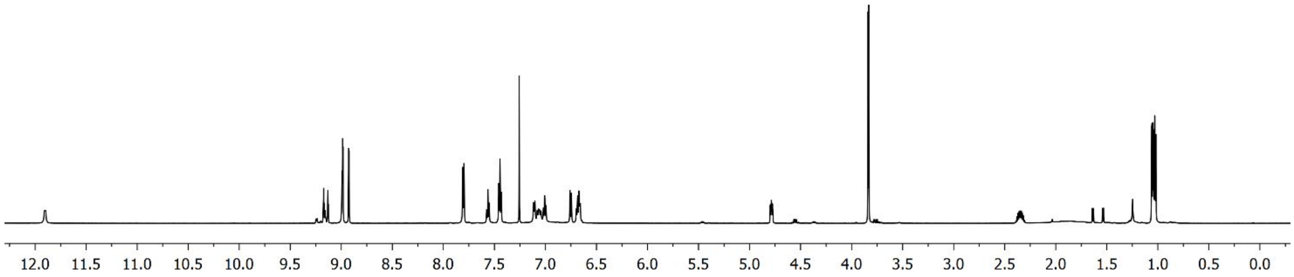

Figure S16. ^{1}H NMR (600 MHz, $CDCl_3$, 25 °C) spectrum of equimolar mixture **2**/**BTDA** (30 mM).

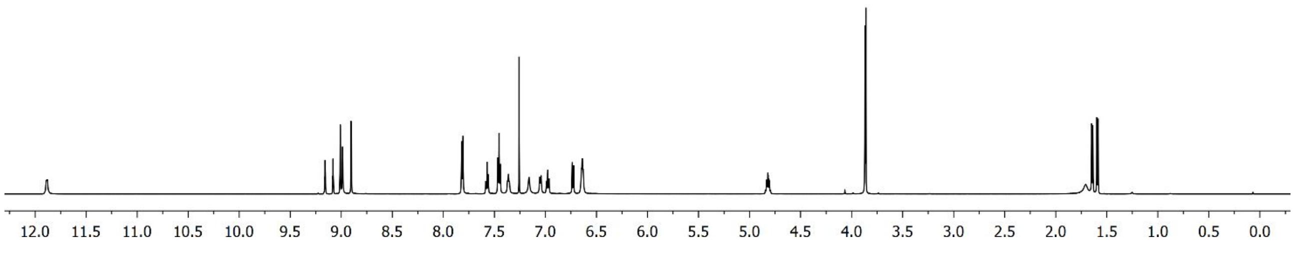

Figure S17. ^{1}H NMR (600 MHz, $CDCl_3$, 25 °C) spectrum of equimolar mixture **3**/**BTDA** (30 mM).

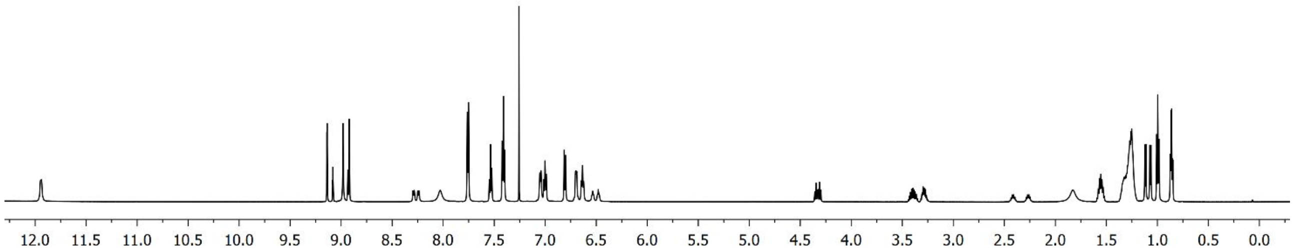

Figure S18. ^{1}H NMR (600 MHz, $CDCl_3$, 25 °C) spectrum of equimolar mixture **4**/**BTDA** (30 mM).

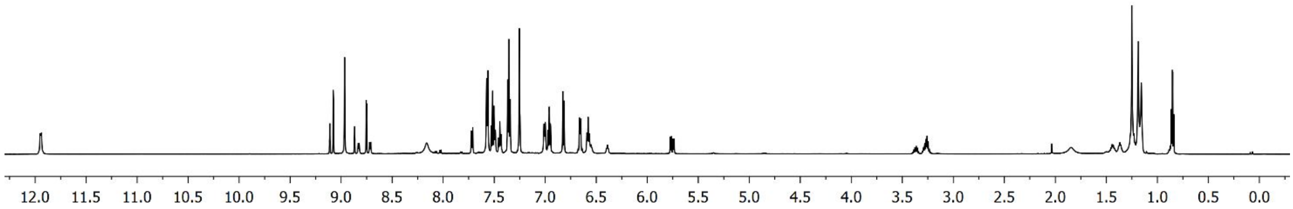

Figure S19. ^{1}H NMR (600 MHz, $CDCl_3$, 25 °C) spectrum of equimolar mixture **5**/**BTDA** (30 mM).

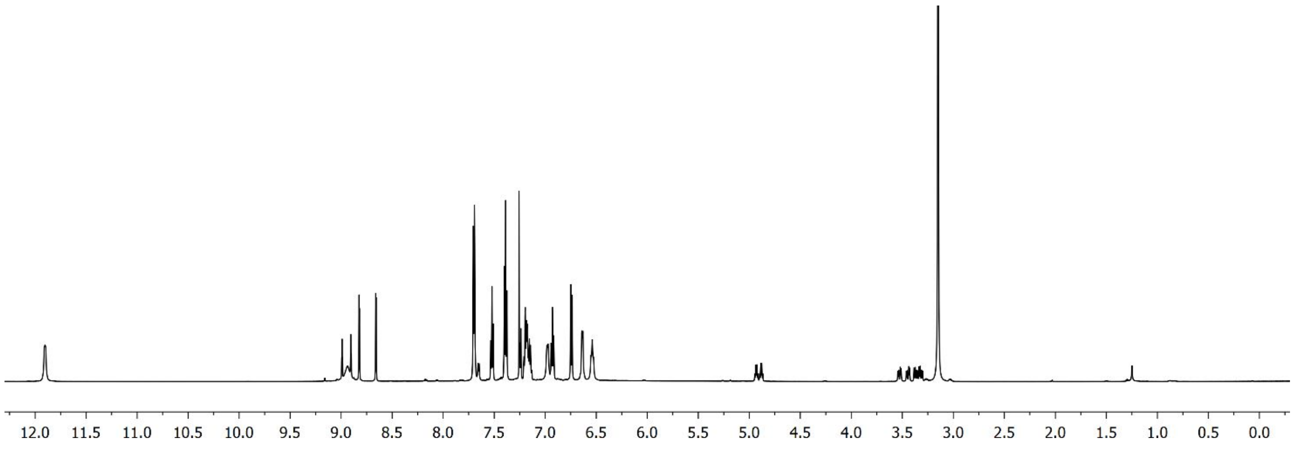

Figure S20. ^{1}H NMR (600 MHz, $CDCl_3$, 25 °C) spectrum of equimolar mixture **9**/**BTDA**/DABCO (30 mM).

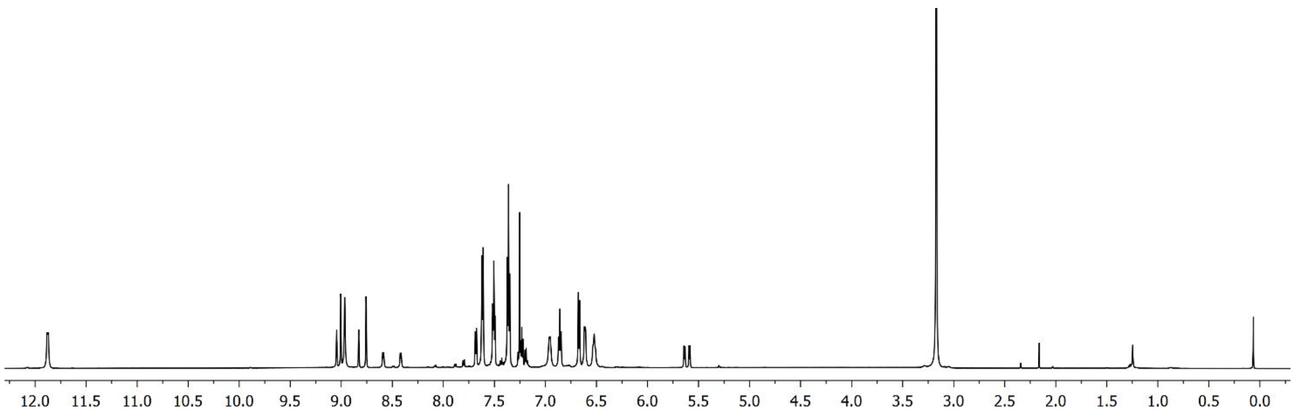

Figure S21. ^{1}H NMR (600 MHz, $CDCl_3$, 25 °C) spectrum of equimolar mixture **10**/**BTDA**/DABCO (30 mM).

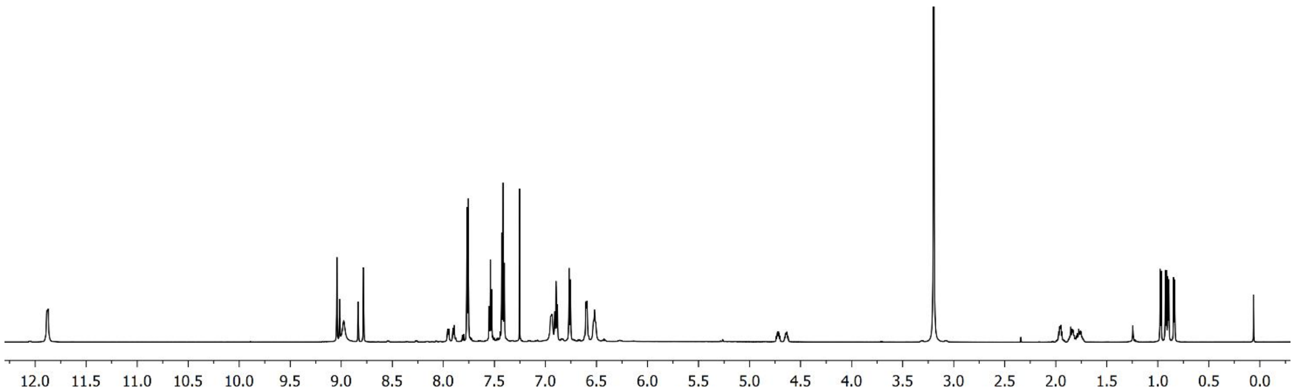

Figure S22. ^{1}H NMR (600 MHz, $CDCl_3$, 25 °C) spectrum of equimolar mixture **11**/**BTDA**/DABCO (30 mM).

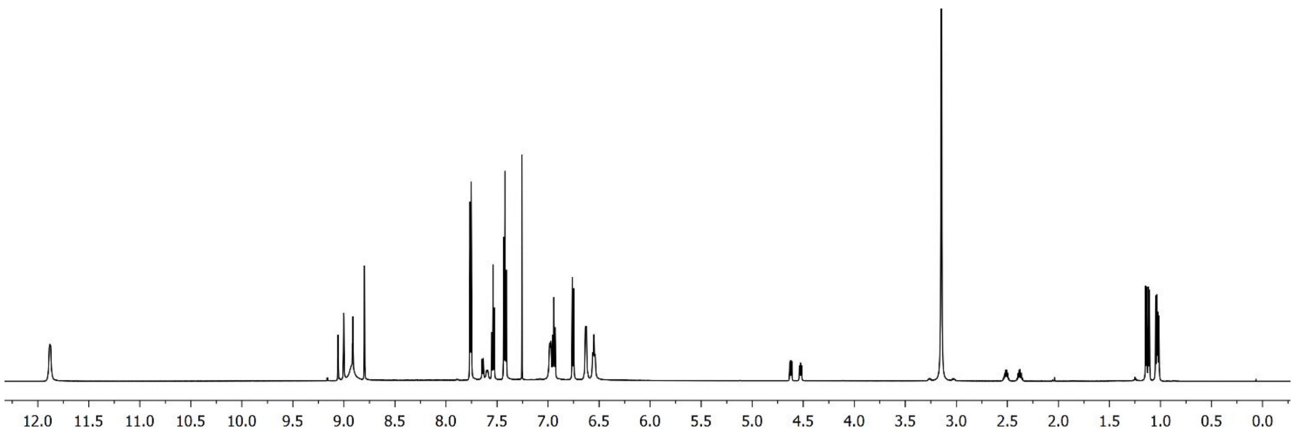

Figure S23. ^{1}H NMR (600 MHz, $CDCl_3$, 25 °C) spectrum of equimolar mixture **12**/**BTDA**/DABCO (30 mM).

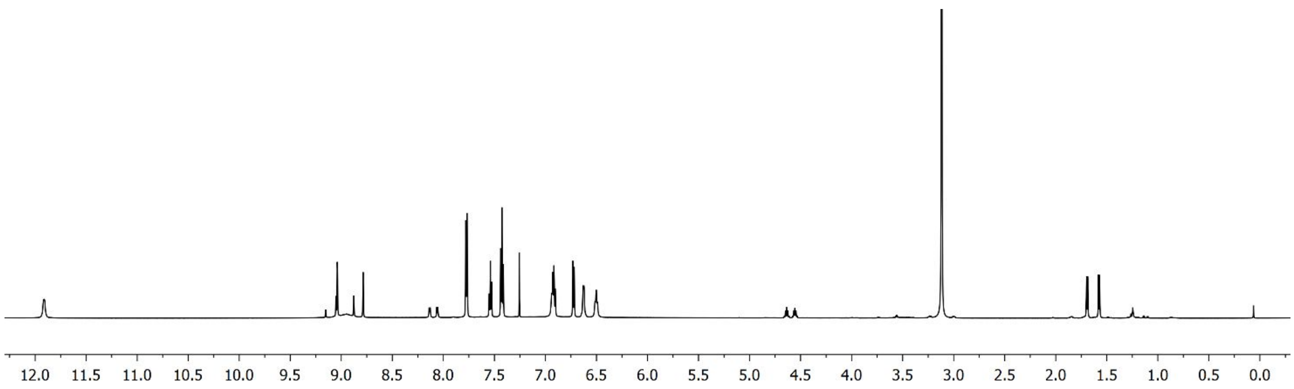

Figure S24. ^{1}H NMR (600 MHz, $CDCl_3$, 25 °C) spectrum of equimolar mixture **13**/**BTDA**/DABCO (30 mM).
